# Spatial and Socio-Classification of Traffic Pollutant Emissions and Associated Mortality Rates in High-Density Hong Kong via Improved Data Analytic Approaches

**DOI:** 10.3390/ijerph18126532

**Published:** 2021-06-17

**Authors:** Hugo Wai Leung Mak, Daisy Chiu Yi Ng

**Affiliations:** 1 Department of Mathematics, The Hong Kong University of Science and Technology, Clear Water Bay, Kowloon, Hong Kong; 2 Department of Geography, The University of Hong Kong, Hong Kong; 3 Department of Electrical and Electronic Engineering, The University of Hong Kong, Hong Kong; 4 Division of Environment and Sustainability, The Hong Kong University of Science and Technology, Clear Water Bay, Kowloon, Hong Kong; ngchiuyi@gmail.com

**Keywords:** traffic NO_x_ and PM_2.5_ emissions, spatial and socio-classification in high density cities, mortality rates and health, urban environment and air quality management, data analytic framework

## Abstract

Excessive traffic pollutant emissions in high-density cities result in thermal discomfort and are associated with devastating health impacts. In this study, an improved data analytic framework that combines geo-processing techniques, social habits of local citizens like traffic patterns and working schedule and district-wise building morphologies was established to retrieve street-level traffic NO_x_ and PM_2.5_ emissions in all 18 districts of Hong Kong. The identification of possible human activity regions further visualizes the intersection between emission sources and human mobility. The updated spatial distribution of traffic emission could serve as good indicators for better air quality management, as well as the planning of social infrastructures in the neighborhood environment. Further, geo-processed traffic emission figures can systematically be distributed to respective districts via mathematical means, while the correlations of NO_x_ and mortality within different case studies range from 0.371 to 0.783, while varying from 0.509 to 0.754 for PM_2.5_, with some assumptions imposed in our study. Outlying districts and good practices of maintaining an environmentally friendly transportation network were also identified and analyzed via statistical means. This newly developed data-driven framework of allocating and quantifying traffic emission could possibly be extended to other dense and heavily polluted cities, with the aim of enhancing health monitoring campaigns and relevant policy implementations.

## 1. Introduction

Outdoor air pollution (OAP) has become a global environmental concern in recent decades, where it exerts challenges and pressure on the atmospheric environment, health expenditures and the normal livelihood of cities. It is also one of the leading factors that constitute to 3.4 million premature deaths each year [1]. In particular, emissions from the transport sector are identified as one of the three major groups of OAP sources within cities, and the associated tailpipe emissions are directly emitted through the exhaust [2,3]. OAP are often connected with deterioration in health quality, premature mortality and economic recession, especially in high-density cities, with respective assumptions [4,5,6]. Kheris (2020) defined Traffic-Related Air Pollution (TRAP) as the ambient air pollution caused by mass traffic activities, i.e., the emission of different kinds of air pollutants (e.g., black carbon, nitrogen oxides (NO_x_), fine particulate matter with a diameter less than 2.5 μm (PM_2.5_), respirable suspended particulates with diameter less than 10 μm (PM_10_) and hydrocarbons) resulting from the use of various types of motorized vehicles, such as passenger cars, motorcycles, and light- and heavy-duty vehicles [6]. Due to the high economic cost and devastating health consequences of TRAP, many developed cities have established strict emission-control policies, implemented emission standards for new vehicles and improved the quality of fuels [7,8]. However, with the increased vehicle usage and roadway expansion in modernized societies [6], as well as the rapid population growth and settlement in developing cities, severe traffic congestions still occur, especially during peak hours, resulting in local air pollution [9] and reduced thermal comfort concerning the atmospheric and the built environment [10].

Based on some environmental assumptions, exposure to traffic-induced OAP can be associated with negative health impacts, from the development of acute symptoms to adverse respiratory diseases that reduce life quality in the long run, as well as the presence of irreversible disruptions to mental health [11,12]. In particular, Chen et al. illustrated the high- and short-term health costs of poor air quality in rapidly urbanizing cities of China, by establishing the possible relationship between exposure to outdoor nitrogen dioxide (NO_2_) and mortality risks of 17 Chinese cities [13]. Moreover, the World Health Organization (WHO) estimated that in 2016, 59% of OAP-related premature deaths were due to ischemic heart disease and stroke, while 18% were due to chronic obstructive pulmonary disease or acute lower respiratory infections, and 6% were due to lung cancer [14]. Nevertheless, the effects of receiving the same amount of ambient pollutant concentration differ among individuals and depend on personal immune system, capabilities of self-recovery, and tolerance to environmental changes within one’s physical body. For a more sensitive individual, a low dosage of pollutant in air will cause immediate unwanted response, while for a less sensitive citizen, intrinsic accumulation will take place, and undesired responses will pop up after a certain time period. Brønnum-Hansen et al. (2018) have shown that, due to the complex mechanisms of emission or pollution components in the atmosphere, exposure–response relationships are generally very weak and hard to be quantified [15], while accurate pollution and exposure assessments require a systematic and precise emission inventory system [16], which also serves as a crucial input for conducting pollution simulations and for better control management [17]. Upon the derivation of pollution figures via modelling techniques, an objective and easily accessed attribute should be taken to link up emissions, pollution figures and potential health impacts, subject to some assumptions related to the surrounding environment. In particular, the Working Group of the European Centre for Environment and Health has considered the use of “mortality rates” for pollution impact assessments [11]—for example, the rates of specific causes of death, cardio-respiratory diseases and chronic non-malignant respiratory diseases [18], as well as lung cancers and selected age-specific deaths [19]. For Hong Kong, due to its data completeness, objectivity and reliability, “mortality rates” obtained from official sources, i.e., based on statistical counts in public and private hospitals, together with medical centres, were adopted as the health assessment attribute, together with other datasets from the Hospital Authority.

The potential environmental and health challenges of excessive traffic emission and associated TRAP depend significantly on liveability conditions, neighboring environmental features and socio-economic status of affected households [20,21], especially in high dense communities like Hong Kong. Thus, it is important to integrate some “social determinants of health” into retrieving fine-scale traffic emission distribution at the first stage, then to investigate the potential connections between citizens, well-being and health quality via air quality modelling [22]. These determinants include social habits of Hong Kong, like working hours, normal peak hours, traffic mobility of citizens, the nature of the district and the respective population density, as well as the building typologies situated at individual streets. Moreover, positive statistical associations have actually been detected between carbon dioxide (CO_2_) emission rates and PM_2.5_ mortality rates in 250 urban cities [23], and between nickel, vanadium, estimated ship emission and cardiovascular mortality in Guangzhou [24]. Brønnum-Hansen et al. (2018) have also explained how traffic NO_x_ emission is positively related to morbidity and mortality in Copenhagen [15]; thus, it is interesting to link up the potential connections between these attributes, with some assumptions made. 

In this study, we focus on developing an improved framework that incorporates the aforementioned social determinants in the cosmopolitan and dense Hong Kong; then, we observe potential connections between district-wise traffic emission estimates and mortality rates, based on the same spatial set-up. Hong Kong is characterized by its high-rise buildings and compact environmental features. Vertical living at highly centralized and mixed land-use built-up areas are commonly found on both neighborhood and metropolitan scales [25]. Such an intensive and multi-functional land-use pattern, on one hand, creates more connective transportation networks and accessibility for daily necessities and promotes efficient spatial planning processes [26], but on the other hand, it obscures privacy and hinders normal wind flow, which leads to poor air ventilation and environmental quality [27]. More critically, due to the concentration of human activities [25], the urban heat island (UHI) effect further traps pollutants and heat energy within street canyons, or the spacing between two neighboring buildings. During the past decade, UHI has become the dominant contributor to increased NO_x_ and PM emissions, as a result leading to higher NO_2_ and PM_2.5_ concentrations in the atmosphere [28,29], especially within commercial and residential districts. It has also been verified that accelerated roadside traffic emissions are closely associated with health problems [30]. Nevertheless, the Transport Department reviewed that even if all proposed emission control strategies are implemented, emissions from road vehicles will likely increase in medium- and high-traffic growth scenarios [31]. By taking into account the “official working hours” of offices and private companies, and the operation timeslots of retail shops in business districts (e.g., commercial areas) of Hong Kong [32], it is expected that the traffic flow between most residential districts and nearby commercial districts will be the heaviest during rush hours (i.e., early morning and from 18:00 HKT to 21:00 HKT); thus, the highest NO_x_ and PM_2.5_ emissions should be detected during these timeslots. As for midnight, while the resulting pollutant concentrations are at low levels due to lower traffic loading, the amount still affects residents living near public roads, especially in Hong Kong, because of tall building typologies near roadside transportation routes [33] under the constraints of limited space for utilization. While the Mass Transit Railway (MTR) network could provide an alternative mode of public transport for citizens, where most of its heavy rail stations have no direct contacts with roadside [34], people still have to transit via MTR stations to working places, dining restaurants and facilities, thus, all citizens of Hong Kong are exposed to certain amounts of TRAP, and associated health risks, no matter the kind of transportation they select or favor.

For the purpose of obtaining a more realistic distribution of district-wise traffic emissions, for the modelling of spatial pollution profiles, which might open new windows for conducting health exposure assessment especially during peak hours, this study attempts to develop a systematic geo-processing framework via data analytic means, which process available hourly traffic NO_x_ and PM_2.5_ emission records of all roads in Hong Kong. To the best of our knowledge, such fine-scale spatial investigations and potential health assessments (with social assumptions imposed) have only been conducted in other countries, like Canada, Poland and the United States [35,36,37], but not in highly dense and populated cities. Therefore, the main objectives, desired results and findings of this study are: (1) To establish an improved and comprehensive framework that combines geo-processing techniques, working norms, peak hours for human mobility, and building morphologies of Hong Kong to retrieve more realistic district-wise and street-level traffic NO_x_ and PM_2.5_ emissions from eight key road transport types; (2) to obtain diurnal variabilities of traffic NO_x_ and PM_2.5_ emissions from each transport type, with the aim of incorporating peak hours into the framework in a more scientific manner; (3) to look for the potential linkages between district-wise traffic emissions and health attributes in different case studies, with respective assumptions outlined in the framework; (4) to identify outlying districts in a highly dense city, then suggest feasible measures for reducing traffic-related health risks and maintaining environmental sustainability and living quality, based on prescribed statistical assumptions. Respective data collection processes and assumptions, as well as the rationale and design of the data-analytic framework, are described in Section 2. Spatial and temporal results and graphs, as well as the statistical analyses, are shown and discussed in Section 3.1, Section 3.2, Section 3.3 and Section 3.4, followed by the possible extensions of current findings. Then, some recommendations and good practices for reducing TRAP and health risks in high-density cities are proposed in Section 4, followed by a short conclusion.

## 2. Data Collection and Methodologies

### 2.1. Study Area

Hong Kong (22°18′10″ N, 114°10′38″ E), officially named the Hong Kong Special Administrative Region of the People’s Republic of China (HKSAR), is considered one of the most developed (i.e., fourth in terms of UN Human Development Index in 2019) and densely populated cities in the world [38]. It is situated in the southern-most coastal area of China, east of Macau, and borders the Guangdong Province via Shenzhen located to its north. Currently, it consists of more than 7.5 million people [39] and has a total land area of 1106.81 km^2^ [40]. Such a dense configuration constitutes a developed transportation network, with the presence of public transport infrastructure and facilities in more than 90% of the land area [41]. Hong Kong has in total three territories, namely the New Territories, Kowloon, and Hong Kong Island. Its 18 individual districts (with each belonging to one of the three territories) are governed by individual district councils. The New Territories is the largest territory (86.2% of the entirety of Hong Kong), and is comprised of the region between Kowloon Ranges and Shenzhen and more than 200 outlying islands, for example, Lantau Island and Lamma Island, as indicated in Figure S1. It also encompasses most hilly terrains and steep slopes situated in the north of Kowloon [42]. Kowloon and Hong Kong Island are separated by the Victoria Harbour, with Kowloon situated directly north; therefore, roadside transportation or ferries are required to transit between the two territories. The well-planned transportation infrastructure within Hong Kong allows transit between the three territories.

Most Hong Kong citizens (3.8 million people) reside in the New Territories, followed by Kowloon (2.2 million people) and the Hong Kong Island (1.3 million people). However, districts in Kowloon generally have higher population densities when compared to Hong Kong Island and the New Territories [43], and 18 districts of Hong Kong are categorized as either residential or non-residential from the Case Study 3 onwards for in-depth analyses. Table S1 shows the detailed population figure, area and population density of each district in 2016, as well as respective individual population growth (in percentage) when compared with the corresponding official figures for 2006 [43], while Figure S2 shows the spatial distribution of the population in all 18 districts of Hong Kong.

### 2.2. Data Collection

#### 2.2.1. Transportation Network and NO_x_ and PM_2.5_ Traffic Emissions of Hong Kong

In this study, only the roadside transportation network within all 18 districts of Hong Kong was considered, but not the railway connection between districts. This is because most heavy railway stations are constructed with an upper cover and are situated far away from main streets. According to a report from MTR in 2016, no measurements of CO_2_ levels within stations and trains exceeded the Level One Criteria of the Environmental Protection Department (EPD) in Hong Kong [44]; thus, indoor air pollution within a railway network might not impose extreme health risks. Further, excessive emissions from different vehicle types on the road were either through direct exhaust (e.g., NO_x_, PM_2.5_ and carbon monoxide (CO)), or via non-tailpipe means (e.g., re-suspended dust and road surface abrasions) [6]. These emissions are dispersed in air and citizens passing the area might easily be exposed to these primary or secondary chemicals and suffer from various health impacts. Based on the available road network dataset in 2015 as released by the Transport Department, there are in total 19,990 road segments in all districts of Hong Kong. Upon alignment of the coordinate system and intersecting with the layer of Hong Kong districts in ArcGIS (Esri, Redlands, CA, USA), 19,266 roads situated within Hong Kong’s boundaries have a geoid length greater than 1 m [45]. Corresponding district-wise data is shown in Table S1. Overall, 9903 road segments (around 1,767,642 m) are situated in nine districts of the New Territories, followed by 5950 road segments (around 681,878 m) in five Kowloon districts, and 4137 road segments (around 451,256 m) are located in four remaining districts of Hong Kong Island.

The territory-wide emission inventories of NO_x_ and PM_2.5_ were obtained from the mobile-source Emission-FACtor-Hong Kong (EMFAC-HK v3.3) model, as developed by EPD [46]. These can be summarized as the product of emission rates and vehicle activity data obtained from the Transport Department. Detailed assumptions and descriptions can be found in [47,48]. Emissions were distributed in accordance with individual roads, and categorized according to vehicle types based on the assumptions outlined in [47]. The processed hourly averaged NO_x_ and PM_2.5_ traffic emission datasets of eight different categories of transportation during the entirety of 2015 were obtained from EPD, HKSAR Government [49]. Upon quality assurance and control (QA & QC), the raw datasets consisted of “CTS_ID” being linked to the road network of Hong Kong, the “road length” (in m), which has to be further analyzed via geo-processing techniques, “hour” (from 0–23 within a day), and “emission figures” (in g per km) of eight transport types, including private cars, taxis, private light buses and non-franchised buses (SPB), van-type light goods vehicles weighing less than 3.5 tonnes (LV), light goods vehicles with two axles (LGV), medium goods vehicles more than 6 m long (MGV + HGV), green and red minibuses (PLB) and buses. It is worth noting that the local government has shifted from diesel fuel taxis to liquefied petroleum gas (LPG) taxis completely since January 2006, with the aim of minimizing only the PM_2.5_ emitted [50]; thus, high NO_x_ emission values remain. Further, as highlighted by EPD, while emissions from road transport decreased by more than 40% from 2001 to 2018, the vehicle kilometers travelled increased by 21% during the same period; therefore, there is a genuine need to analyze emission trends, identify major emission sources, and implement appropriate air quality management strategies that could possibly alleviate health challenges afterwards. While vehicles that belong to the category “private car” took up 40–70% of total traffic flow in most major roads of Hong Kong [51], the amount of traffic NO_x_ and PM_2.5_ emissions were less than for MGV + HGV and buses. After collocating the “CTS_ID” with that in the road network and Hong Kong map layers, traffic emission records of 19,223 and 19,282 road segments were made available for NO_x_ and PM_2.5_, respectively. The exact number of roads considered in each case study depends on environmental and geographical constraints and criteria (in Section 3.3). Table S2 shows the detailed numerical figures and information.

#### 2.2.2. Building and Podium Attributes of 18 Districts

**Figure 1:**
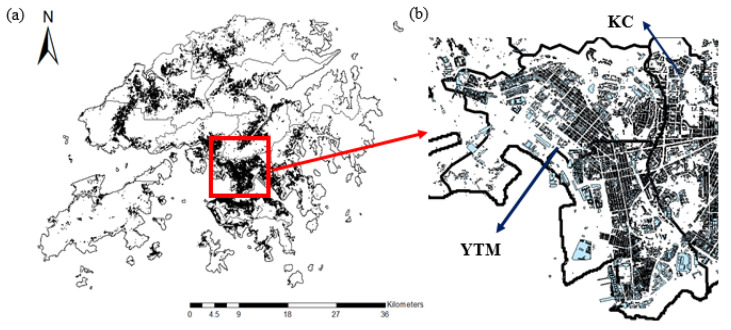
(**a**) The spatial distribution of buildings and podiums within 18 districts of Hong Kong; (**b**) the enlarged top plain view of the red square box in Figure 1a—precisely the Yau Tsim Mong (YTM) District and part of the neighboring Kowloon City (KC) District. Buildings and podiums are shaded in pale blue.

To investigate how realistic spatial configuration at the street level could alter traffic emissions of districts, which might link with the potential health impacts caused under the prescribed assumptions, the HK Building and Podium datasets from the Buildings Department and Survey and Mapping Office (SMO), Lands Department, HKSAR Government, were taken into consideration in Case Study 4. This aims to identify active human activity regions within the district, and eliminate roads that have minimal intersections between human movement/activity regions and emission occurrence. By combining available raw building and podium layers via ArcGIS, there are in total 143,496 infrastructure units in all districts, with their geo-referenced code, name of building, podium or facility, and height of structure clearly shown in the combined dataset. Such a dataset precisely reflects the locations in which mass activities were taking place, for example working places, dining places, and recreational and sports facilities. Figure 1a shows the spatial distribution of buildings and podiums in Hong Kong, and Figure 1b shows the top plain view of the Yau Tsim Mong (YTM) district and part of Kowloon City (KC) in its neighborhood. YTM is one of the most densely populated districts in Hong Kong, with its population density similar to that of the entirety of Kowloon. It is observed from Figure 1a that most buildings and podiums of Hong Kong are concentrated and distributed within Kowloon and Hong Kong Islands, in contrast with the low density within the New Territories. This is generally in line with the higher population densities of these districts (shown in Table S1). 

#### 2.2.3. Health Datasets: Mortality Rates of TRAP-Related Diseases 

In Hong Kong, there is no direct database that counts the number of patients being physically affected by TRAP, and some citizens may not be aware of the deterioration of health quality within the short term. Even being affected, some may be reluctant to seek medical advice. Despite such shortcomings, district-wise mortality figures of all related diseases were obtained from an official source in Hong Kong, that is HealthyHK, Department of Health, HKSAR Government [52], and selected as health attributes. 

The figures of death statistics shown on this official governmental site were categorized by the International Classification of Diseases (ICD). By means of “mortality rates” as comparable to previous studies [11,15,18,19], we refer to “confirmed death cases of individual disease per 100,000 population” in each district. It is well noted that, for conducting in-depth health impact assessments, pollutant concentration figures have to be obtained, either from measurements or modelling techniques, while it is well justified that precise emission profiles will likely lead to accurate pollutant distribution and respective health status. Thus, we attempt to trace any statistical relationships between district-wise NO_x_ and PM_2.5_ emissions and mortality rates under different case studies, with some assumptions imposed. While the results still consist of unavoidable uncertainties, this may provide insights for alleviating TRAP in the foreseeable future, while in-depth health exposure studies will also be conducted at the next stage. 

Major diseases that could lead to both direct and indirect mortality, especially for PM_2.5_ in ambient environments, were taken into consideration. This includes respiratory cerebrovascular and chronic diseases for the former group, and diabetes and pneumonia for the latter group. Standard medical names and ICD codes of these available datasets on the HealthyHK website are as shown in Table 1. For calculating “mortality rates”, the averaged rate from 2015–2018 (latest available period on website) were adopted, based on several assumptions: (1) The figures must be representative, and any uncertainties and potential bias within a particular year should have been removed; (2) some diseases have an incubation period even after their outbreak, and long-term health risks could be important; (3) a complement of the research gaps of “missing induction time” in previous literature [53]; (4) the effects of infectious diseases, for example, immediate mortality caused by COVID-19 infection should be minimized and averaged out. Thus, the “mortality rate” figure of each disease in our study is the temporal average of computed mortality rates within the four-year period. Detailed numerical figures due to individual related disease are as shown in Table S3. 

### 2.3. Underlying Assumptions and Potential Bias

Several environmental and scientific assumptions have been made throughout this study, which enabled the incorporated emission, road and district-wise data to become a surrogate of population exposure, for example, assuming constant external emission sources, limited effective spatial region of traffic emissions, prescribed chemical lifetime of pollutants, and uniform spatial distribution of traffic emissions. Further, a potential source of bias with regard to data interpretation and linkage is provided. These assumptions minimized the uncertainties of the study, at least within the Hong Kong domain.

#### 2.3.1. Necessary Assumptions for conducting Spatial Assessment

(a) Constant External Emission Sources

To isolate the effects from other emission sources and to focus on traffic-induced emissions, it is assumed that emission factors of all other natural and anthropogenic sources and forces (except road transportation) are kept constant within the emission inventory. Potential sources of NO_x_ and PM_2.5_ emissions include public electricity generation, civil aviation, navigation, combustion in construction sites and power plants [49], as well as non-combustion sources like dusts from paved roads and construction, and extreme climate events like hill fires, which generate PM_2.5_ and PM_10_. Thus, we assume that there were no “net effects” caused by other emission sources.

(b) Lifetime and Effective Spatial Region of Traffic Emissions

Based on previous literature and modelling results, the lifetime of NO_x_ is several hours near the ground surface, and is around 1–2 weeks in the upper troposphere [54]. The discrepancy of the lifetime within different parts of the atmosphere can be attributed to the difference in the rate constant of chemical reactions, together with the amount of hydroxyl group (OH) in the upper troposphere and boundary layer. From modelling perspectives, the lifetime of NO_x_ is around 5–6 h during summer, mainly because of the abundance of OH and RO_2_ concentrations within our atmosphere. This also leads to the shortened NO_x_ lifetime during daytime [55]. However, due to the reduction of aerosol concentration, the corresponding lifetime of NO_x_ increases at night [55]. As for PM_2.5_, it can travel for a longer distance within the lower part of the atmosphere when compared to NO_x_ and stay in air for hours to weeks [56]. These tiny particles may easily affect respiratory and circulatory systems [57]. Moreover, outdoor PM_2.5_ levels are elevated in calm wind circumstances [58], or in the absence of precipitation within the troposphere [59]. Thus, it is first assumed that pollutant emissions during the entire day are connected with environmental and health qualities. Such assumptions were gradually relieved, so that the more realistic emission scenario could be established (see Section 2.4). Regarding the effective spatial region of both NO_x_ and PM_2.5_, while they can easily travel from one district to another before halting their motion in air, the effective distance of both chemicals is assumed to be kept within the boundary of the respective district, for the sake of local statistical analyses.

(c) Uniform Spatial Distribution of Traffic Emissions

After merging the HK District and road network datasets from the Transport Department, it was found that some roads cross through two or more districts, and most of them are long roads (in terms of geoid length). However, as the amount of traffic emission for each vehicle type was arranged and assigned according to CTS_ID, there is only one set of hourly averaged data available for the individual road. Thus, it becomes vital to develop a consistent and accurate mathematical approach to distribute emission figures among districts. Here, both NO_x_ and PM_2.5_ traffic emissions are assumed to be evenly distributed within the road, on spatial and temporal scales. The detailed calculation is outlined in Section 2.4, and Figure S3 shows an illustration based on Yen Chow Street West (CTS_ID: 915130).

#### 2.3.2. Potential Ecological Fallacy of Data Interpretation and Uncertainties 

Some potential bias and uncertainties may arise in this study. In particular, different age groups have different probabilities of suffering from diseases listed in Table 1. Due to deteriorated cardiovascular functionalities [60], elderly people generally have higher risks of suffering from cardiovascular diseases and myocardial infarction [61,62], and are more vulnerable to PM pollution in ambient environments, which possibly leads to cardio-respiratory mortalities in the long run [63]. Moreover, maternal prenatal exposure to PM pollution before giving birth and during the first trimester of pregnancy may also lead to increased infant mortality and cardiac risks during childhood [64,65]. Second, elderly people of Hong Kong may easily suffer from one of the three chronic diseases [66], or other treatment-related complications. Mortality can also be attributed to the combination of causes, rather than one specific disease type. For the sake of statistical analyses, and to “by-pass” the complexities of atmospheric “disturbance”, it is assumed that the attained traffic emissions are positively related to modelled air pollution, which is then associated with health challenges, as validated in previous studies as aforementioned. Further, people within the district will have equal probabilities of suffering from unfavorable health impacts induced by traffic-related pollution.

### 2.4. Methodologies

#### 2.4.1. Overall Framework of Case Studies 1 and 2

In this framework, Case Study 1 accounts for all available NO_x_ and PM_2.5_ emission figures (see Section 2.2.1), without imposing any spatial or temporal selection. The rationale is to obtain an overall picture of emission distribution within an individual district of Hong Kong. Then, ongoing case studies attempt to trace potential spatial changes and variabilities of emission profiles in different districts after a particular spatial or temporal constraint has been incorporated into the geo-processing framework.

**Figure 2:**
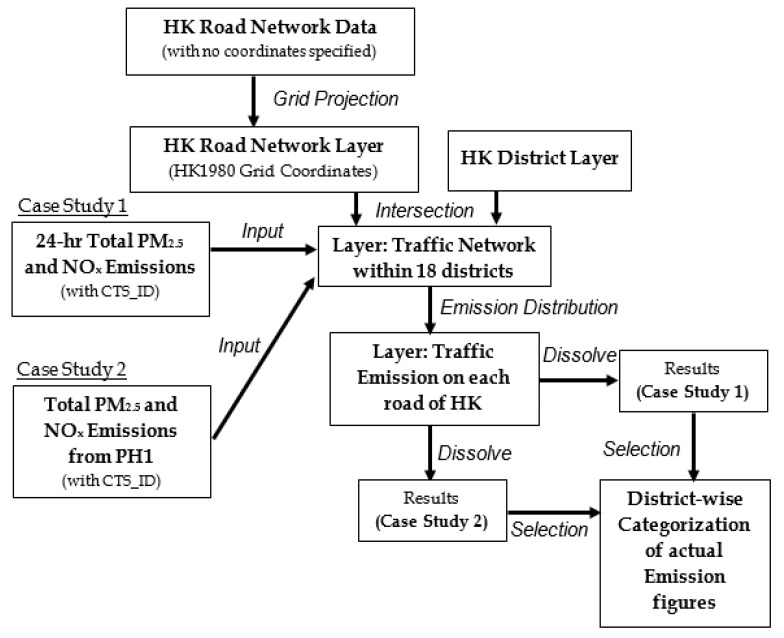
Framework and flowchart (indicated by the direction of arrows) of Case Studies 1 and 2, starting from grid projection to obtaining emission profiles. Descriptions in italics represent either the operation or geo-processing procedures within ArcGIS. HK: Hong Kong; PH1: The first defined period of peak hours.

It has been verified that mass vehicular emissions in Atlanta, Georgia during peak hours could increase up to 17% [67], while the health risks constituted by on-road emissions during morning rush hour period could be 20–45% higher than the rush hour period in the afternoon, as validated in the United States [68]. Thus, the concept of “peak hours” (PHs) has become important because emissions during these hours could cause huge health impacts to citizens. In Case Study 2, the first period of peak hours (PH1) is defined based on diurnal variations of traffic emissions from all eight vehicles types (see Section 3.2 for details), so that the busiest period of a day can be better identified. Case Studies 3 and 4 take account of selected social factors, like the nature of districts and building typologies (see Section 2.4.2 and Section 2.4.3). Figure 2 illustrates the overall framework and flowchart of data handling, geo-processing techniques adopted within the ArcGIS platform, and mathematical techniques for distributing traffic NO_x_ and PM_2.5_ emissions among streets. These processes are common for all case studies, and all available layer files in ArcGIS share a uniform coordinate system, that is, the Hong Kong 1980 Grid (Transverse Mercator) coordinate system. The road network and district layers were compiled by the “Intersect” geo-processing function, so that they became geo-referenced for further data integration. The processed 24-hr total NO_x_ and PM_2.5_ emissions as derived from the EMFAC-HK model were ingested into the GIS dashboard, and were distributed to each of the 18 districts, based on the procedures below. This, on one hand, accounts for the phenomenon as described in Section 2.3.1, with one long road crossing through two or more districts, and on the other hand, avoids the over-estimation of traffic emission within an individual district. 

First, assuming that emissions spread uniformly over each district and street, the Inverse Distance Weighting (IDW) method was applied to estimate the emission of any point in between two spatial locations with known emission values. Suppose the emission values of positions *X* ($E_{X}$) and *Z* ($E_{Z}$), as well as the distances *XY* $\left(d_{1}\right)$ and *YZ* $\left(d_{2}\right)$ are, respectively, known, the emission figure at position *Y* ($E_{Y})$ can be calculated by Equation (1). A pictorial representation of IDW is as shown in Figure S4. The actual lengths of all road segments (with geoid length > 1 m) were computed by the “calculate Geometry” dialog box in ArcGIS; then, the ratio (R) was computed as in Equation (2). Finally, the adjusted emission figure of each road segment ($E_{A}$) situated within only 1 district, or the portion that belongs to a particular district, was then calculated using Equation (3), through multiplying the original emission figure ($E_{o}$) by *R*.

(1) $$E_{Y}=\frac{E_{X}\cdot d_{2}+E_{Z}\cdot d_{1}}{d_{1}+d_{2}}$$

(2) $$R=\frac{d_{A}}{d_{G}}$$

where $d_{A}$ is the actual length of the road segment, $d_{G}>1\;m$ represents the geoid length obtained from the original road network dataset.

(3) $$E_{A}=R\cdot E_{O}$$

Table 2 shows an illustration of the entire procedure and respective numerical quantities of Yen Chow Street West (CTS_ID: 915130) (See Figure S3). Next, the “dissolve” geo-processing function was applied to solve another problem arising from the raw road network data: There may be two or more records (ID in GIS) for the same road in the original dataset, for example, the Tsing Long Highway (CTS_ID: 915276) situated at Yuen Long, a district in the New Territories. After the “dissolve” adjustment, each individual road that does not cross over two or more districts will only have one single record, with *R* = 1.

Finally, within the “show table” window in ArcGIS, the corresponding NO_x_ and PM_2.5_ emissions of all roads in each district were obtained with the “select by attributes” function, via the selection of the desired district. The total emission values of each vehicle type ($E_{V_{1}},\;E_{V_{2}},\ldots ,E_{V_{8}})$ could then be summed up, and the traffic pollutant emission (*E*) of a particular district was further summed up as in Equation (4), where *N* is the number of roads in that district, and $E_{A_{j},V_{i}}$ represents the pollutant emission of the *j*th road due to vehicle type *i*.

(4) $$E=\overset{8}{\underset{i=1}{\sum }}E_{V_{i}}=\overset{8}{\underset{i=1}{\sum }}\overset{N}{\underset{j=1}{\sum }}E_{A_{j},V_{i}}$$

#### 2.4.2. Case Study 3: Categorization of Nature of Districts and Refinement of Peak Hours (PH2)

According to the PH definition from the Traffic Survey and Support Division, Transport Department [69], the PHs of Hong Kong are from 7 to 10 am and 4 to 7 pm, which is in line with the working hours of the Government Office (08:30–17:30 HKT) and most private companies (09:00–18:00 HKT) of Hong Kong [32]. On top of this, as outlined in Chen et al. (2003) and Han et al. (2020), urban–rural heterogeneity of vehicular emissions is associated with pollution effects [70,71]; thus, it is important to group districts of a similar nature (i.e., residential or non-residential) in Hong Kong for respective assessments. Table S1 shows the population percentage of each district in Hong Kong, ranging from 2% (Islands and Wan Chai) to 9% (Kwun Tong and Sha Tin) [43]. In particular, seven districts with a population percentage of 6% or above are placed in Group 1—residential districts (i.e., mainly for residential use or new towns); while the remaining 11 districts belong to Group 2—non-residential districts (i.e., mainly commercial, industrial or mixed-land use). Group 1 (population percentage) consists of Sha Tin (9.00%), Yuen Long (8.29%) and Tuen Mun (6.68%), as well as Kwun Tong (8.85%), Eastern (7.57%), Kwai Tsing (7.10%) and Sai Kung (6.30%) districts.

In Case Study 3, we also make a slight refinement of PHs as defined in [69] by further incorporating two assumptions: (1) Many employees will buy daily necessities and do window-shopping after finishing work; (2) the chemical lifetime of traffic pollutants in air is around several hours (see Section 2.3.1). PH2 is refined as 5–9 am and 2–8 pm (inclusive) for Group 1 districts, and 5–10 am and 2–7 pm (inclusive) for Group 2 districts. Here, 12-h traffic emission data were extracted for both district groups for fair analyses, and it is reasonable to assume that the majority of the working population depart from home at around 7–9 am, and stay in the office from 9 am (or 10 am) till 5 pm (or 6 pm), then return back home at around 6–8 pm in the evening. Hence, Case Study 3 has also considered both citizens’ habits and footprints of respective districts, and could possibly provide more realistic emission profiles.

#### 2.4.3. Case Study 4: Incorporation of Building Typologies

On top of UHI effects as aforementioned, it was verified that building types and architectural designs could have impacted the residential outdoor and indoor PM levels, as observed in New York City [72], and also in high-density cities like Hong Kong [73]. Thus, additional emphasis should be placed on the consideration of building and infrastructural layers in each district. Hence, Case Study 4 attempts to first identify road segments that surround residential and common human activity areas within each district to realize the distributions of TRAP in a more practical manner.

To achieve this goal, two geo-processing steps were conducted in ArcGIS on top of the established framework, namely (1) application of the “union” function to combine building and podium layers (see Section 2.2.2); (2) usage of the “buffer” function concerning the combined layer of (1), with a buffering distance of 0.2 km (representing potential human activity region surrounding facilities). The exact number of remaining roads of each district is shown in Table S2. Finally, the emission figures obtained based on Case Study 3 were directly mapped to the buffered layer.

#### 2.4.4. Relationship between Traffic Emissions and Health Measures of Hong Kong 

As mentioned in previous sections and studies, district-wise traffic emissions were related to pollution, while exposure to pollutants are positively related to mortality rates. Some case studies in other countries also established the linear relationship between traffic emissions and mortality parameters [74,75,76]; thus, statistical methods are adopted here to look for their potential connections in Hong Kong, subject to the assumptions made and the potential uncertainties induced. Here, we implicitly assume a linear relationship between the two quantities, and apply least-square regression to obtain all correlation plots. Then, the Pearson coefficient (*R* value), Root-Mean-Square Errors (RMSE), slope of the best-fit line, and *p* value of regressed models were adopted for statistical comparisons. Case Study 4 incorporated many social factors, like potential human mobility and traffic patterns, peak hours, building morphologies, district nature and population density; thus, the regressed equations obtained in Case Study 4 were adopted to compute the vertical distances between all data pairs and their nearest neighborhood on the best-fit line, then to identify the possible outlying districts (see Section 3.4). Moreover, geographical or socioeconomic-specific reasons that cause such statistical mismatch were also discussed.

## 3. Results and Discussion

### 3.1. District-Wise Traffic NO_x_ and PM_2.5_ Emissions and Mortality Rates of Hong Kong 

**Figure 3:**
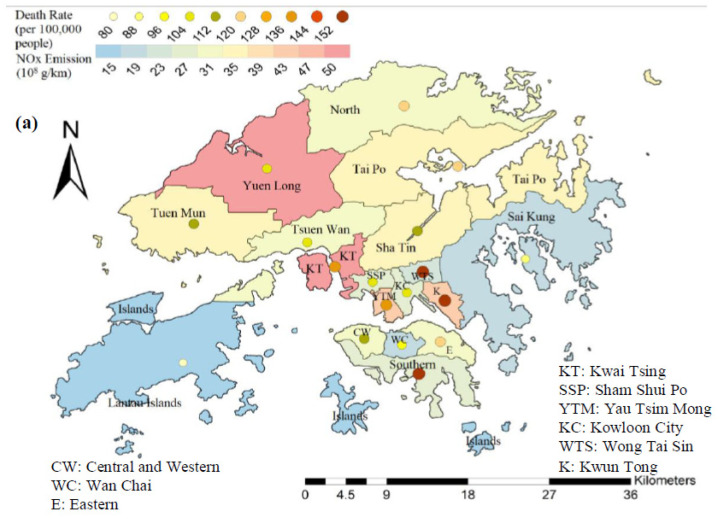

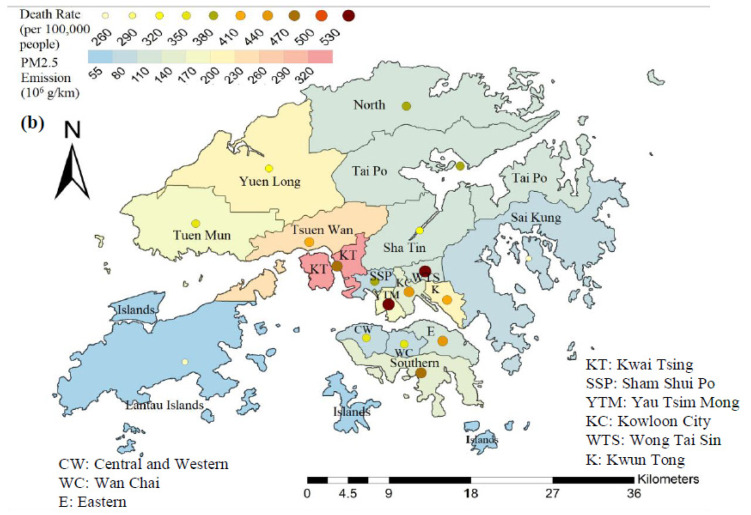
District-wise (**a**) traffic NO_x_ (10^8^ g/km) and (**b**) PM_2.5_ (10^6^ g/km) emissions in Hong Kong (Case Study 1), together with mortality rates (per 100,000 people) superposed (as circles).

One of the goals of this study is to obtain street-level fine scale traffic NO_x_ and PM_2.5_ emissions resulting from eight key road transport types, and their potential connections with respective health risks, based on prescribed assumptions. Figure 3 shows the spatial maps of traffic NO_x_ and PM_2.5_ emissions in all 18 districts, based on the results of Case Study 1 and the framework of Figure 2. Mortality rates (per 100,000 people) are also superposed on each district, shown in the form of filled-in circles. 

Overall NO_x_ emission figures in 2015 ranged from 1.14 × 10^9^ g/km in Islands to 5.03 × 10^9^ g/km in Kwai Tsing, with a district-wise average of 2.99 × 10^9^ g/km, while the overall district-averaged PM_2.5_ emission figure was 1.29 × 10^8^ g/km; the range was from 2.84 × 10^7^ g/km (Islands) to 3.11 × 10^8^ g/km (Kwai Tsing). There are also some interesting observations: (1) For districts with lower NO_x_ emissions, Islands, Sai Kung and Wan Chai had equivalently low mortality rates, while Southern and Wong Tai Sin had excessively high mortality rates, which implicates that there were still other fatal sources of NO_x_ emissions (apart from traffic), which were converted into NO_2_ concentrations within these districts; (2) for districts with relatively high NO_x_ emissions, Yau Tsim Mong, Kwai Tsing and Kwun Tong possessed similarly high mortality rates; however, a relatively low death rate was detected in Yuen Long, a new residential town in the New Territories; (3) Kwai Tsing had the highest PM_2.5_ traffic emissions (50% above Tsuen Wan) and placed third in associated mortality rate, positioned after Wong Tai Sin and Yau Tsim Mong; (4) two relatively remote districts in the New Territories—Islands and Sai Kung had low PM_2.5_ emission and mortality rates, followed by districts with mixed land use, like Sha Tin and Tai Po.

Spatial discrepancy of emission figures appeared because MGV + HGV and bus emissions were generally 4–5 times higher than other vehicle types, while 45.9% and 51.9% of NO_x_ and PM_2.5_ emissions in Kwai Tsing were due to MGV + HGV, as compared to the Hong Kong average (27.2% and 31.2%, respectively). On top of this, PLB generally accounted for approximately 5% of NO_x_ emissions (in the order of 10^8^ g/km) and 12.5% of PM_2.5_ emissions (order of 10^7^ g/km) in most districts; however, Islands had no PLB emissions, thus having the least amount of NO_x_ and PM_2.5_ emissions. It is interesting to note that the amount of NO_x_ emission was more than 10 times higher than PM_2.5_, which can be attributed to the unusually high levels of roadside NO_2_ emitted by franchised buses and liquefied petroleum gas (LPG) vehicles, as well as the installation of particulate filters within these vehicle types [77]. For example, as shown in Table S4, no PM_2.5_ emissions were recorded for taxis, but constituted around 5% of NO_x_ emission during 2015. Such an effect was much more obvious in commercial districts, like Central and Western (14.4%), Wan Chai (12.5%) and Kowloon City (10.3%). On top of this, the NO_x_ emission figures remained high throughout the year because, at that time, many owners of taxis and minibuses had not adopted the use of cleaner fuels and environmentally friendly devices, while a huge amount of NO_2_ was emitted whenever the catalytic converters of these vehicle types became defective [77]. Detailed numerical figures of emissions and mortality rates of each district are as shown in Table S5. 

**Figure 4:**
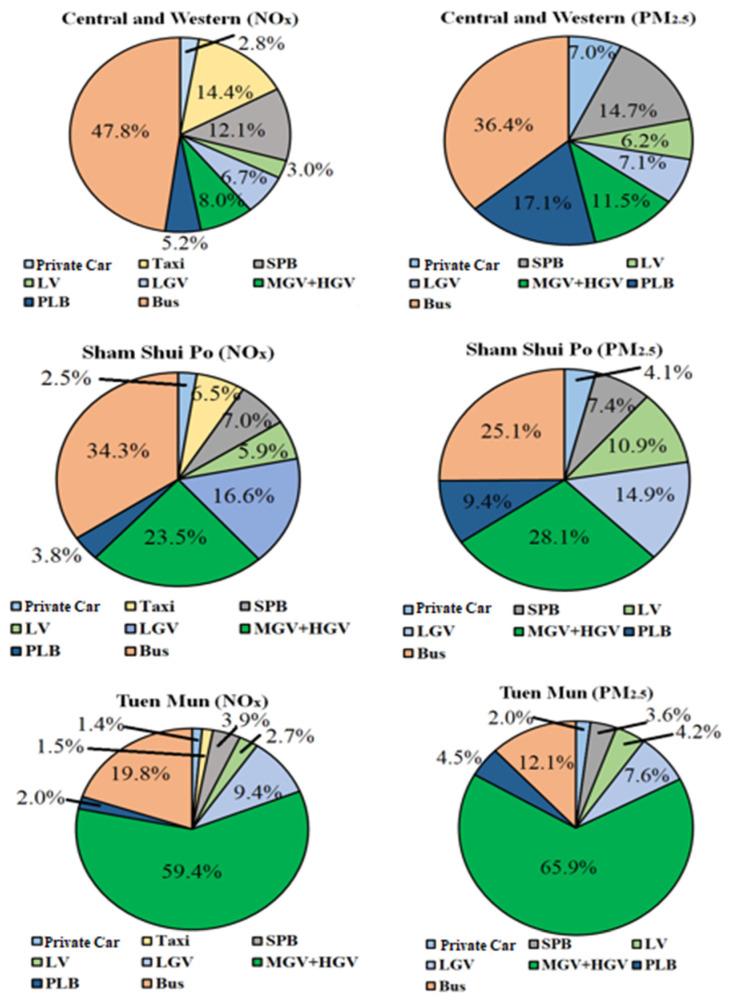
Percentages of NO_x_ and PM_2.5_ emissions from different vehicle types in Central and Western (commercial district), Sham Shui Po (industrial district) and Tuen Mun (residential district). SPB: private light buses and non-franchised buses, LV: van-type light goods vehicles weighing less than 3.5 tonnes, LGV: light goods vehicles with two axles, MGV + HGV: medium goods vehicles more than 6 m long, PLB: green and red minibuses.

Districts of Hong Kong can generally be categorized into three major types, as well as mixed types (i.e., the combination of two or three of these three district types), namely commercial, industrial, and residential districts. With the aid of understanding the discrepancy of percentages of emissions due to different vehicle types, Figure 4 shows the respective distributions of NO_x_ and PM_2.5_ emissions in Central and Western (commercial type), Sham Shui Po (industrial type) and Tuen Mun (residential type). The distributions were totally different from each other, where buses dominated both NO_x_ (47.8%) and PM_2.5_ (36.4%) emissions in Central and Western, followed by taxis (for NO_x_) and SPB (for both pollutants). For Sham Shui Po, buses and MGV + HGV contributed almost equal portions of emissions for both pollutants, which added up to 57.8% of NO_x_ and 53.2% of PM_2.5_ traffic emissions within the district. The proportion of SPB in industrial districts (~7%) was relatively less than that in commercial districts (~13%), while LGV contributed a significant portion of emissions (~15%) for both pollutants. Finally, MGV + HGV was a dominant contributor (around 60%) of both pollutants for residential districts (with a population of >6%), followed by buses (~15%), then LGV (~8%). The contribution of all other five vehicle types was of minimal influence. Percentages of vehicle-type emissions of all other districts are as shown in Table S4. The different sources shown in the pie charts, together with spatial variations of traffic emissions, highlight the importance of incorporating land-use patterns, citizens’ travel habits and human activities into the developed framework. Using this as a potential reference, monitoring of TRAP, reduction of health exposure to harmful traffic pollution, and better infrastructural designs of the built environment can be effectively planned.

### 3.2. Diurnal Variations of Transportation Emissions

**Figure 5:**
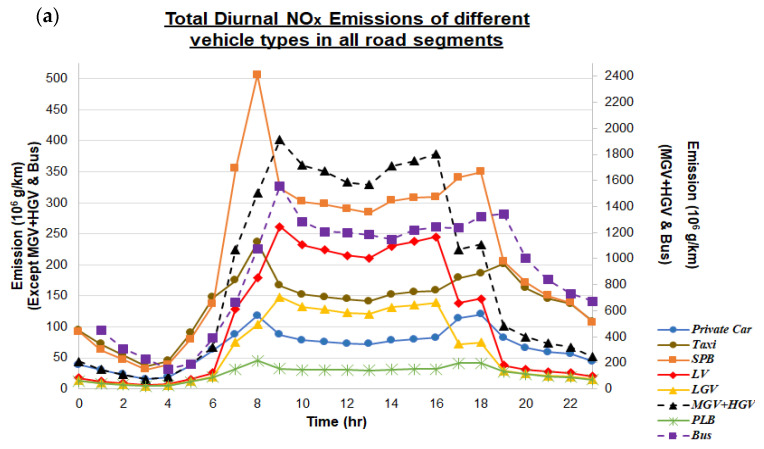

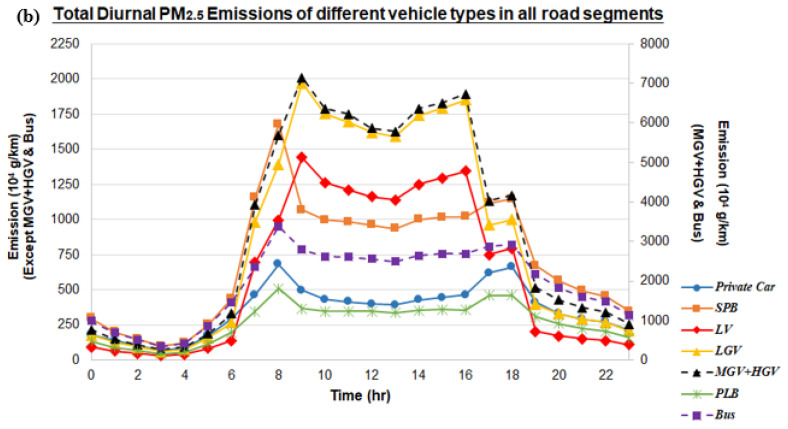
Diurnal Trends of (**a**) NO_x_ (10^6^ g/km) and (**b**) PM_2.5_ (10^4^ g/km) emissions observed for eight vehicle types within 2015 at all road segments with available traffic emission records. The right color bar is applicable for MGV + HGV and bus, while other six vehicle types adopt the color bar on the left side.

Our next goal focuses on acquiring diurnal variabilities of traffic NO_x_ and PM_2.5_ emissions for each transport type. Thus, the total NO_x_ and PM_2.5_ emissions of each vehicle type within every hour and every road segment with available emission record were summed up, and could serve as a reference for assessing the actual impacts of traffic-generated emissions via combining with modelling tools. Figure 5a,b show the diurnal trends and variations of traffic emissions in 2015. Since the emission values of MGV + HGV and bus were much higher than the other six vehicle types, two different color bars were adopted. For NO_x_, emissions of all vehicle types reached their peaks either at 8 am (PLB, private car, SPB, taxi) or 9 am (bus, LGV, LV, MGV + HGV), while similar temporal features can also be observed for PM_2.5_ (bus, PLB, private car, SPB at 8 am, and LGV, LV, MGV + HGV at 9 am). Moreover, most temporal graphs showed double peaks, one in the morning, and another at 4 pm, 6 pm or 7 pm, which correspond to the travelling period of most of the working population in Hong Kong. It was observed that the afternoon peak hours of LGV, LV and MGV + HGV were earlier than other vehicle types, because these vehicles are mainly responsible for transmitting massive, fragile and heavy goods from one district to another, and usually these goods have to reach offices or residential places before 6 pm (closing hours of many offices in Hong Kong). On the other hand, other vehicle types mainly carry passengers back home, or to shopping and dining places; thus, their peak hours are slightly delayed.

Diurnal cycle of traffic emissions does not only alter the chemical composition and dispersion within the boundary layer, especially near ground [78], the complex mixture of induced pollutants within horizontal and vertical layers, and governed atmospheric chemistry transports also alter the roadside dispersion of emission contents [79], which could potentially lead to uneven pollutant distribution and associated health effects, or even mortality in an exaggerated manner. Based on the temporal trends shown in Figure 5, the PH1 of Case Study 2 includes all available emission records obtained at 7 am, 8 am, 9 am, 5 pm and 6 pm (i.e., the 5-h period). Results show that around 33.4% of NO_x_ emissions and 32.8% of PM_2.5_ emissions occurred during these 5-h period, while the raw NO_x_ emission of Case Study 2 ranged from 3.39 × 10^8^ g/km (29.7% of Case Study 1) in Islands to 2.00 × 10^9^ g/km (39.8% of Case Study 1) in Kwai Tsing. The “peak-hour effect” was less obvious for the PM_2.5_ case, which ranged from 7.25 × 10^6^ g/km (25.5% of Case Study 1) in Islands to 7.30 × 10^7^ g/km (23.5% of Case Study 1) in Kwai Tsing. Moreover, the sources of traffic emissions from different vehicle types at the three district types were similar to those of Case Study 1; however, the portion of both NO_x_ and PM_2.5_ emissions from SPB significantly increased. This is mainly due to the higher traffic flow of private light buses during peak hours for carrying children to schools, and the working population between residential flats and large-scale companies, universities and industrial hubs. 

### 3.3. Street-Level Traffic NO_x_ and PM_2.5_ Emissions under Different Case Studies and Connection with Mortality Rates of Hong Kong under Prescribed Assumptions

This section fulfills the goals of obtaining more practical spatial distributions of traffic emissions via the improved data analytic framework, which may provide feasibility to connect with district-wise mortality rates of Hong Kong, under the assumptions stated in Section 2.3.

First, after the application of the “buffering” function discussed in Section 2.4.3, the number of roads being considered decreased to 12,500, while the number of roads within nine districts of the New Territories decreased by an average of 45.9%, as compared to 19.2% in Hong Kong Island and 27.1% in Kowloon. This could be attributed to the uneven district-wise land-use distribution, with more land in the New Territories allocated as agricultural land, fish ponds and water bodies [80], which barely have people passing through.

**Figure 6:**
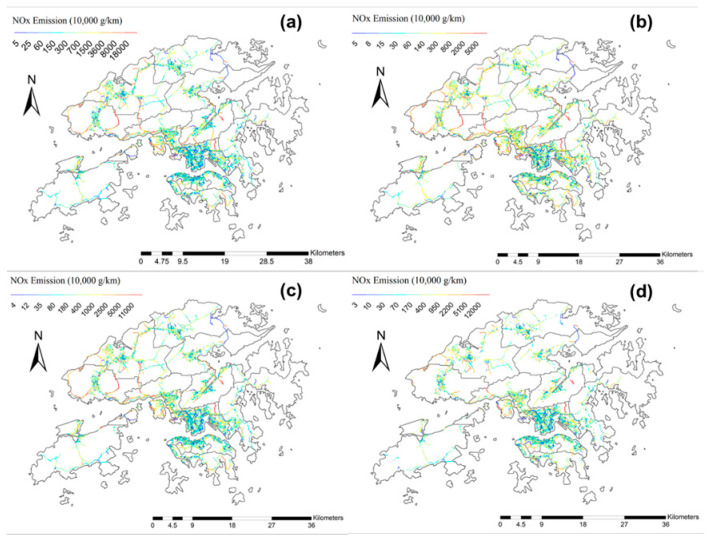

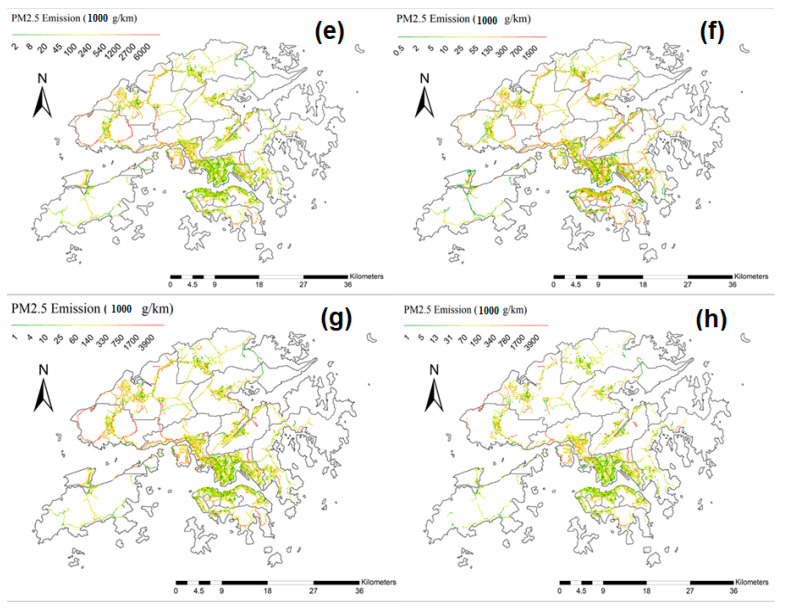
Spatial Distributions of Street-Level NO_x_ (Top Panel: (**a**–**d**)) and PM_2.5_ (Bottom Panel: (**e**–**h**)) Traffic Emissions (in 10^4^ g/km and 10^3^ g/km respectively) retrieved from (**a**) and (**e**) Case Study 1; (**b**) and (**f**) Case Study 2; (**c**) and (**g**) Case Study 3; and (**d**) and (**h**) Case Study 4 within all districts in HK. In (**d**) and (**h**), the 0.2 km buffering distance of human activity regions was applied.

Figure 6 shows the spatial distributions of NO_x_ and PM_2.5_ traffic emissions in Hong Kong, from Case Study 1 to Case Study 4, with different criteria like PHs (PH1 and PH2), social habits and morphologies being incorporated into the framework (see Figure 2). The color bars are all arranged in geometric progression. 

The “relative” NO_x_ emissions of all districts were of similar spatial characteristics within all four case studies, with generally higher numerical values in the New Territories, as compared to Kowloon and Hong Kong Island. There are two highlighted changes in spatial features: (1) Relative NO_x_ in North and Sha Tin had an obvious increase starting from Case Study 2 onward, which indicates the occurrence of massive traffic flow during the PH1 period in these two “northern” districts; (2) after the application of the “buffer” function for road selection in Case Study 4, some roads with originally high NO_x_ emissions in Tuen Mun, Southern, as well as the boundaries in between Tsuen Wan, Tuen Mun and Yuen Long were no longer considered, which indicates that the emissions observed at these roads were actually not within the neighborhood of infrastructure systems, and thus may not be exposed to humans. The extraordinarily high traffic emissions could partly be attributed to the LV, LGV, MGV + HGV, which carry food and necessities from Shenzhen Bay to Hong Kong. On the other hand, the road segments with low NO_x_ emissions in the North also disappeared from the streets considered in Case Study 4, which could possibly explain the slight decrement from Case Study 3 to Case Study 4. Due to the existence of many water bodies and mountainous areas in the New Territories, the corresponding number of roads in these districts had a sharp decline upon buffering. In particular, only 821 road segments in Yuen Long were considered in Case Study 4, as compared to 1835 road segments in Case Study 1. For PM_2.5_, the spatial characteristics and changes among different case studies were similar to those of NO_x_, which indirectly confirms the significance and purposes of individual case study designs. More than that, obvious increases in PM_2.5_ emissions (in a relative sense) were detected in many places within Case Study 2, for example, North, Sha Tin and Tai Po in the New Territories, the southern region of Kowloon, Wan Chai and the Central and Western districts, with many road segments indicated in red. Such observation highlights the increase in traffic flow between residential districts and commercial districts during mornings and late afternoons. Similar to NO_x_, after buffer selection, some road segments of Sha Tin and Tuen Mun, as well as the intersecting roads between Tsuen Wan and Yuen Long, were not included in Case Study 4. This further implicates that, though high levels of traffic emissions were detected in these road segments, the impacts on normal living of citizens may not be immediate, because many of these roads are actually situated far away from activity regions. Interestingly, abrupt fluctuation in PM_2.5_ emissions was detected in Tuen Mun West, and first became less obvious in Case Study 2, but then resumed relatively higher emissions in Case Study 3, as compared to other districts. Such spatial discrepancy occurred because of different PH definitions. Seven more hours in early morning (5 am and 6 am), early afternoon and evening (2–4 pm and 7–8 pm) were further included in the definition of PH2, which verifies that traffic might not be concentrated within PH1 hours in Tuen Mun. This is because Tuen Mun is situated far from the centre and commercial hubs of Hong Kong; therefore, the working population has to allocate more time to daily travelling, i.e., departing from home earlier in the morning, while returning home later than people residing in other districts of Hong Kong.

**Figure 7:**
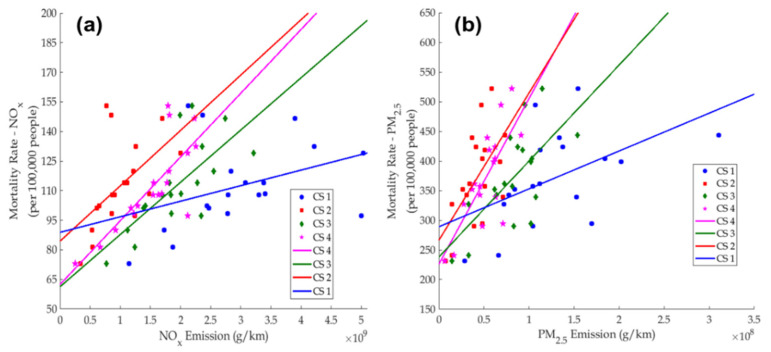
Potential Connection between Mortality Rate of (**a**) NO_x_ versus respective Emissions (10^9^ g/km), and (**b**) PM_2.5_ versus respective Emissions (10^8^ g/km) in all four case studies in Hong Kong districts, with assumptions and uncertainties of Section 2.3 taken into account, together with the corresponding best-fit line obtained by least square regression techniques. Mortality figures are per 100,000 people and based on all disease types as shown in Table 1, while the symbols of each case study (CS) are as stated.

Despite the existence of uncertainties, and the fact that statistical conclusions may only be limited to the Hong Kong region, the potential connection between district-wise emission figures and mortality rates was evaluated, based on the logical assumption and deduction stated and argued in Section 2.3. Respective correlations and best-fit lines between raw or updated NO_x_ and PM_2.5_ emission figures in 2015 and district-wise mortality rates are as shown in Figure 7a,b. Here, each data point represents one of the 18 districts in Hong Kong, and the corresponding best-fit line was obtained via least-square regression. Positive correlations between the two quantities could be observed in all four case studies. However, when all raw emission figures were included without considering PHs nor building types and typologies (Case Study 1), the correlation was least obvious. After taking into account PHs within the geo-processing framework (Case Studies 2 and 3), the statistical associations became more obvious, with points being less scattered and gradually nearing the resulting best-fit line. For NO_x_, the *p* values of Case Study 2 onwards were all less than 0.05, while all *p* values satisfied such a constraint for PM_2.5_. It is worthwhile to mention that, based on our assumptions in this study, out of all disease types (see Table 1), 61.4% of mortality causes from NO_x_ emission were categorized as malignant neoplasm of trachea, bronchus and lung (C33–34), while the same disease type only accounted for 18.6% in the case of PM_2.5_ emissions. The second most obvious disease type (24.8%) of NO_x_ emissions is chronic lower respiratory diseases (J40–47), and pneumonia (J12–18) is highly associated with PM_2.5_ emissions (40.5%) in Hong Kong (not shown).

By considering all assumptions and potential uncertainties, the key statistical parameters of each case study, in terms of all 18 districts and the division of districts into two groups (see Section 2.4.2) are as shown in the Table S6. The overall *R* values of both NO_x_ and PM_2.5_ increased from 0.371 to 0.783 for NO_x_, and from 0.509 to 0.754 for PM_2.5_ after advanced geo-processing, PHs and morphological constraints were applied. The corresponding RMSEs and *p* values decreased accordingly. It is interesting to note that, upon division of district types in Case Study 3, the two individual best-fit lines (in Group 1 and 2 districts) exhibited a relatively clearer trend when compared with the overall regressed output. However, after road selection was applied in Case Study 4, the *R* value (RMSE) of residential districts (Group 1—with only seven data pairs) was slightly less (higher) than that of all 18 districts, for both NO_x_ (by 0.023) and PM_2.5_ (by 0.037). Nevertheless, the connection between emissions and mortality figures of non-residential districts (Group 2) was clearer, with enhanced *R* value and lower RMSE, especially for PM_2.5_ (0.889 in Case Study 3 and 0.958 in Case Study 4 in terms of *R* values). There are two potential conclusions and insights: (1) The updated emission inventory could offer references for air pollution modelling, as well as the development and implementation of policies with regard to public health maintenance of Hong Kong; (2) The more direct connection between emissions and mortality in non-residential districts of Hong Kong could be attributed to the setting up of greening facilities and barriers in many residential districts, which likely alleviates devastating health impacts induced by excessive traffic emissions. Overall, this improved data analytic framework and the incorporation of different constraints could effectively update street level emission inventories and provide more realistic, reliable and accurate traffic emission figures in the long run, especially for high density and mixed land-use cities like Hong Kong. On top of this, differences between *R* values and RMSEs in different case studies (as shown in Table S6) have shown respective importance of individual social determinants of health, for example, the selection of PHs, and the identification of building and infrastructure systems in each district.

### 3.4. Identification of Outlying Districts and Spatial Features

This section attempts to make good use of the improved framework to identify districts that have either extraordinary traffic emissions or excessive mortality rates. In our established framework, obvious connection between the two quantities could be observed in Case Study 4 after geospatial, social and morphological factors were incorporated into the model’s development, subject to several uncertainties as mentioned in Section 2.3.2. Here, the regressed equations of NO_x_ and PM_2.5_ in Figure 7 were adopted as references for the purpose of “predicting” mortality rates and identifying potential outlying districts within Hong Kong, which could also be extended to other high-density cities. A similar approach was used to associate PM_2.5_ in air with the increased risk of asthma for new born babies [74]. 

In this context, the percentage of deviation (PD) based on the linear equation was defined as in Equation (5), where ${\mathrm{MR}}_{P}$ and ${\mathrm{MR}}_{A}$ represent the predicted and actual mortality rates, and $E_{{\mathrm{NO}}_{X}}$ and $E_{{\mathrm{PM}}_{2.5}}$ are the adjusted NO_x_ and PM_2.5_ emission figures obtained in Case Study 4. The slopes and *y*-intercepts of both equation of straight lines are based on the pink lines of Figure 7a,b.

(5) $$\mathrm{Percentage}\;\mathrm{of}\;\mathrm{deviation}\;(\mathrm{PD})\;(\%)=\frac{{\mathrm{MR}}_{P}-{\mathrm{MR}}_{A}}{{\mathrm{MR}}_{A}}\times 100\%$$

where ${\mathrm{MR}}_{P}=\{\begin{array}{c} 3.23\times 10^{-8}\cdot E_{{\mathrm{NO}}_{X}}+62.43\\ 2.79\times 10^{-6}\cdot E_{{\mathrm{PM}}_{2.5}}+226.32 \end{array}$.

The averaged PD $\left({\mathrm{PD}}_{A}\right)$ and the standard deviation (SD) of this statistical sample (with *N* = 18) were computed, so that the two thresholds could be laid down, with one being 1 SD higher, and another being 1 SD lower than ${\mathrm{PD}}_{A}$. The districts with PD not lying in the range of $\left({\mathrm{PD}}_{A}-\mathrm{SD},{\;\mathrm{PD}}_{A}+\mathrm{SD}\right)$ are considered as outlying districts. For NO_x_ (PM_2.5_), ${\mathrm{PD}}_{A}$ and SD were −1.20% and 11.15% (−1.94% and 14.84%), respectively. Therefore, the corresponding range of non-outlying districts was (−12.36%, +9.96%) for NO_x_, and (−16.79%, +12.90%) for PM_2.5_. After these two intervals were obtained, three outlying districts could be identified for NO_x_, and six for the case of PM_2.5_. Table 3 shows the summary of all outlying districts for each pollutant. Exact PDs of individual districts are provided in Table S5. 

Concerning the first district group (with excessively high mortality rates), the Southern district consists of many factories and industrial buildings, especially in the Ap Lei Chau and Wong Chuk Hang areas, which emit high amounts of NO_2_ and PM through combustions and daily industrial activities. On top of this, the harbor in Aberdeen of the Southern district consists of many ferries, which increases the amount of SO_2_ in the lower atmosphere. The effects of excessive concentrations of different pollutants add up and lead to different health impacts, or even long-term mortality. As for Wong Tai Sin, it is considered as one of the “oldest” districts of Hong Kong, with a lot of elderly people, public housing estates and ageing infrastructure, but without proper and appropriate greening plans implemented within residential areas and surrounding environments. In particular, the Wong Tai Sin Temple is a well-known shrine that emits smoke every day [81], which could affect the socioeconomic and health status of residents within the district. For the Yau Tsim Mong district, Mong Kok consists of numerous urban street canyons that trap pollutants and restrict proper air ventilation, as a result causing local thermal discomfort and inducing long-term mortality [82]. 

In contrast, the second district group (with excessive traffic emission, but “relatively” less mortality) consists of new towns like Tuen Mun and Yuen Long. Despite the close proximity of residential areas to major traffic routes, greening policies and recreational facilities have recently been incorporated into urban planning and renewal of these districts. Further, citizens residing in new towns are usually more energetic and have healthier lifestyles, for example, active participation in physical activities based on prescribed action plans [83], and the maintenance of the “getting rest and waking up earlier” practice, due to the spatial location. On top of this, traffic emissions that take place in highways of new towns may not have caused serious health impacts to citizens, because most of them are usually situated far away from human activity regions, as reflected in Case Study 4 (shown in Table S2).

### 3.5. Discussion and Limitations

The spatial results obtained via our improved data analytic framework, and the potential connection between TRAP and health risks within this study are generally comparable to previous literature. For example, the major mortality cause of excessive NO_x_ emission in our study is malignant neoplasm (61.4%, C33–34 of Table 1), rather than other respiratory mortality causes like chronic lower respiratory diseases (24.8%, J40–47 of Table 1). This is in line with the findings of Ho et al. (2020), who conducted a territory-wide register-based study and confirmed that neighborhood NO_2_ causes cancer-related mortality more easily than respiratory mortalities [84]. Moreover, as remarked by [85], the vehicle kilometres travelled (VKT) of Hong Kong’s traffic network is more closely associated with natural-cause and cardiovascular mortalities of elderly people when compared to respiratory mortality. Our results obtained in this study are quite similar after imposing different scientific assumptions, where traffic-PM_2.5_ emissions have higher impacts on ischaemic heart diseases (11.0%, I20, I23–25 of Table 1) than respiratory infections and diseases (3.56% after summing up). Regarding the selection of the human activity region in Case Study 4, our buffering distance (0.2 km) was also comparable with the conclusion of Special Report Number 17 released by the Health Effects Institute, that a distance of up to 300 to 500 m from a major road is identified as an exposure zone due to TRAP [86]. Moreover, the potential connection between neighborhood traffic emissions (Case Study 4) and mortality is the same as the conclusion obtained in [87], where the addition of a vertical component to the existing two-dimensional exposure model (i.e., the model becomes three-dimensional) actually enhances the association between both NO_2_ and PM_2.5_ and health-related risks, especially for the elderly population group. This opens a new window for applying our data-analytic framework for traffic emission monitoring and related modelling studies.

While the understanding of the Hong Kong scenario has been enhanced, there may still be some practical uncertainties. First, cross-boundary effects and pollutant flow dynamics have not yet been considered, and our assumption has removed the interactivity of pollutants between neighboring districts. Therefore, future goals lie on the governing of temporal mobility patterns of residents in a city, studying the potential confounding of different geospatial and socio-economic factors and respective changes, the prediction of exposed pollution patterns via the combination of agent-based modelling, machine learning and big data analytics [88,89,90]. Further, atmospheric and chemistry-transport models can also provide a fine-scale spatial and temporal distribution of meteorological quantities and vertical profiles of pollutants, which can be combined with datasets of various data structures [91,92,93]. Thus, the complicated and changing connections between three parameters, namely, traffic emissions, traffic-induced pollution (or exposure), and mortality rates, can be fully interpreted. Modelling outputs can also enrich our understanding of chemical processes that actually happen within the atmosphere. In particular, the more realistic traffic emissions obtained in this study (Case Study 4) can further be incorporated into different models for health studies.

Apart from air pollution, different socio-economic factors and statuses among districts can further be considered and studied, for example, lifestyles and habits, demographic effects, and vulnerability of different age groups to traffic-induced OAP. Moreover, other emission contributors of NO_x_ and PM_2.5_, the incubation period of pollution-induced diseases and lagging effects of mortality can also be taken into consideration, so that better prediction and assessment of pollution exposure can be achieved via data-analytic means. With ample data on hand, traffic will no longer be categorized as the only emission source, while the connections between different emission sources should have been better established. Overall, the framework developed within this study can easily be generalized and extended to other cities, together with the incorporation of different socio-economic datasets, similar to this paper. The entire framework can also be applied to study emission patterns of other chemicals, for example, SO_2_, and greenhouse gases like CO_2_ and CH_4_ [94], which can be combined with modelling approaches to analyze the actual impacts of emissions on the urban environment and public health status. 

## 4. Recommendations 

To reduce the associated human health impacts caused by emissions originating from existing transportation network and infrastructure, several practical means are suggested as follows and are aligned with the concept of “sustainable city development”. These include: (1) Implementation of a sustainable and green urban transport system, especially in highly polluted districts or cities, for example, the adoption of electric vehicles, motorcycles and hybrid cars, which do not contain any dangerous gas emissions, and at the same time conserve energy and can possibly reduce fuel consumption [95]. Such innovative green technologies will ultimately create a healthier and more sustainable community, as a result lead to better life quality from the socio-economic point of view; (2) Promotion of the use of zero emission franchised buses during power generation processes [96], which eventually encourages complete usage of environmentally friendly fuels, and renewable energy resources for electricity generation. Such a plan has been on the list of Hong Kong for several years, but has not yet been successful; therefore, future goals should target at reducing the dependence of non-renewable energy sources, while at the same time installing low-cost sensors at roadside and ambient monitoring stations to obtain more comprehensive pollutant attributes, then releasing instantaneous environmental information to the public according to open data initiatives [97]. Environmental impact assessment and the identification of traffic emission hotspots can also be conducted via the enhanced monitoring network; (3) Provision of better management of public transportation allocation and working arrangements within high density context of Hong Kong, which can also be extended to other metropolitan cities, like Singapore. In particular, the transport departments of respective cities can reduce the allocation of specific vehicle types during peak hours, as identified via the observation of temporal trends, while large-scale companies and businesses should encourage flexible timeslots of getting off from work to minimize overcrowding of people on streets, thus improving the overall health conditions of the community. Further, the public road transport, like bus and mini-bus networks, numbers and routes should be reorganized and restructured, so that the newly developed transport network could result in shorter travel time and easy interchanges, which bring less harm to human health, in both local and regional scales.

## 5. Conclusions

With the application of different geo-processing and gridding techniques, scientific identification of peak hours (PH) and the consideration of urban morphologies, this study has practically quantified the spatial distribution of traffic NO_x_ and PM_2.5_ emissions within individual streets of Hong Kong, which is one of the most densely populated cities in the world. The updated street level emission inventory obtained via this framework could provide references for conducting health assessments, and could potentially improve modelling performance and capabilities, especially in meteorological or air pollution retrievals. Out of the social determinants being considered in this study, the selection of PHs is useful for estimating NO_x_ emissions, while both PH selection and identification of human activity paths are equally important in obtaining PM_2.5_ emission estimates. This study also attempts to trace potential connection between traffic emissions and mortality rates of Hong Kong, based on prescribed environmental and theoretical assumptions. In terms of statistics, *R* values of the linearly regressed lines between the two parameters increase upon the incorporation of more spatial constraints and social habits, and are accompanied with the decrease in RMSEs and *p*-values. Based on the designed “percentage of deviation” mechanism and emission inventory retrieved in Case Study 4, a total of three and six outlying districts could be found for the cases of traffic NO_x_ and PM_2.5_, respectively. With further investigation and analysis, it was found that the discrepancies are attributed to two different reasons: (1) Architectural design features like greening have already been implemented in these new towns, or (2) the possibility of having other major pollutant emission sources within concerned districts. Overall, this newly developed geo-processing and data-analytic framework is extremely useful for incorporating different social determinants, and eventually updates emission inventories at street scales in a highly urbanized city. The resulting spatial emission profiles could enhance and facilitate modelling performance, then connect with different kinds of spatial assessments and policy implementations related to health perspectives, thus, a city with effective environmental control strategies, sustainability and a healthy neighborhood environment can be developed in the foreseeable future.

## Figures and Tables

**Table 1:** Types of diseases (ICD codes) * considered for the calculation of mortality rates of each district (time period: 2015–2018).

Related to PM_2.5_ Traffic Emission	Respiratory Tuberculosis (A15–16) Malignant neoplasm of trachea, bronchus and lung (C33–34) Diabetes mellitus (E10–14) Other ischaemic heart diseases (I20, 23–25) Pulmonary embolism (I26) Cerebrovascular diseases (I60–69) Remainder of diseases of the respiratory system (J00–06, J30–39, J60–98) Pneumonia (J12–18) Other acute lower respiratory infection (J20–22) Chronic lower respiratory diseases (J40–47)
Related to NO_x_ Traffic Emission	Respiratory Tuberculosis (A15–16) Malignant neoplasm of trachea, bronchus and lung (C33–34) Remainder of diseases of the respiratory system (J00–06, J30–39, J60–98) Chronic lower respiratory diseases (J40–47)

* Source: https://www.healthyhk.gov.hk/phisweb/enquiry/mo_ysad10_e.html (accessed on 2 March 2021).

**Table 2:** Distribution of NO_x_ emissions among Sham Shui Po and Yau Tsim Mong. (Yen Chow Street West, CTS_ID: 915130).

ID in GIS	District	CTS_ID	$d_{G}$ (m) *	$d_{A}$ (m) *	*R*	Private Car Emission (g/km) *	Taxi Emission (g/km) *
	Entire Street	915130	82.0047			7101.74	39,327.33
1176	Sham Shui Po	915130	82.0047	70.5128	0.8599	6106.52	33,816.11
1183	Yau Tsim Mong	915130	82.0047	11.4920	0.1401	995.23	5511.27

* Values of $d_{G}$ and $d_{A}$ are corrected to 4 decimal places, while values of private car and taxi emission figures are corrected to 2 decimal places.

**Table 3:** Outlying districts identified based on the “percentage of deviation” mechanism, for NO_x_ and PM_2.5_ traffic emissions, respectively.

	Mortality Rate >> Traffic Emission in “Relative Sense” (Percentages)	Mortality Rate << Traffic Emission in “Relative Sense” (Percentages)
NO_x_	Southern (+18.22%); Wong Tai Sin (+21.47%)	Yuen Long (−34.71%)
PM_2.5_	Southern (+14.25%); Wong Tai Sin (+15.42%); Yau Tsim Mong (+13.32%)	Sha Tin (−24.24%); Tuen Mun (−18.19%); Yuen Long (−44.35%)

## Data Availability

The data presented in this study are available on request from the corresponding author.

## Supplementary Materials

The following are available online at https://www.mdpi.com/article/10.3390/ijerph18126532/s1, Table S1: Official demographic and socio quantities of 18 districts in Hong Kong, based on available official sources, with units indicated in brackets and year of attainment afterwards, Table S2: Exact number of roads in each district within Case Studies 1–4, based on geo-processing procedures in ArcGIS platform, and further spatial considerations, for assessments of NO_x_ and PM_2.5_ emissions respectively, Table S3: Averaged death rates of each district (per 100,000 people) in terms of diseases induced by NO_x_ and PM_2.5_ traffic emissions. The respective column heading represents the International Classification of Diseases (ICD) codes (Time Period: 2015–2018), Table S4: Percentages of NO_x_ and PM_2.5_ emissions of each vehicle type within each district of Hong Kong, based on Case Study 1 (i.e., original traffic emissions without adjustments nor incorporation of social characteristics), Table S5: Traffic NO_x_ and PM_2.5_ emissions and mortality rates (per 100,000 people) in different case studies, and the percentage of deviation (PD) as calculated by Equation (5) of the main text, Table S6: Comparison of statistical parameters of correlation plots between mortality and emission figures of NO_x_ and PM_2.5_, by assuming linear regression and least-square fitting holds. Numerical figures in brackets are corresponding statistical parameters for Group 1 districts (G1—residential districts, *n* = 7) and Group 2 districts (G2—non-residential districts, *n* = 11) respectively, in that order, for Case Studies 3 and 4, Figure S1: Three territories of Hong Kong (i.e., New Territories, Kowloon, and Hong Kong Island) and surrounding areas, with the position of the Hong Kong International Airport included, as extracted from Hong Kong Hopping site, Figure S2: Population figures of 18 districts in Hong Kong, Figure S3: Example of handling long roads within Hong Kong (based on Section 2.3.1 (c)), Figure S4: Illustration of Inverse Distance Weighting (IDW) method in calculating traffic emission at position *Y*, which is in between two points, *X* and *Z*, with known emission figures.
