# Supplementary material for: Spatial and Socio-Classification of Traffic Pollutant Emissions and Associated Mortality Rates in High-Density Hong Kong via Improved Data Analytic Approaches
Source: Int J Environ Res Public Health. 2021 Jun 17;18(12):6532. doi: 10.3390/ijerph18126532 (PMC8296480; doi:10.3390/ijerph18126532)
Supplement: Supplementary file 1 [file ijerph-18-06532-s001.zip › ijerph-1199221-supplementary.pdf]

Supplementary Information

Spatial and Socio-Classification of Traffic Pollutant Emissions and Associated Mortality Rates in High-Density Hong Kong via Improved Data Analytic Approaches

Hugo Wai Leung MAK [1,2,3,*] and Daisy Chiu Yi NG [4]

[1] Department of Mathematics, The Hong Kong University of Science and Technology, Clear Water Bay, Kowloon, Hong Kong Special Administrative Region.

[2] Department of Geography, The University of Hong Kong, Hong Kong Special Administrative Region.

[3] Department of Electrical and Electronic Engineering, The University of Hong Kong, Hong Kong Special Administrative Region.

[4] Division of Environment and Sustainability, The Hong Kong University of Science and Technology, Clear Water Bay, Kowloon, Hong Kong Special Administrative Region.

*Correspondence: hwlmak@ust.hk

Table of Contents

Table S1. Official demographic and socio quantities of 18 districts in Hong Kong, based on available official sources, with units indicated in brackets and year of attainment afterwards

Table S2. Exact number of roads in each district within Case Studies 1 – 4, based on geo-processing procedures in ArcGIS platform, and further spatial considerations, for assessments of NO_x and $PM_{2.5}$ emissions respectively

Table S3. Averaged death rates of each district (per 100,000 people) in terms of diseases induced by NO_x and $PM_{2.5}$ traffic emissions. The respective column heading represents the International Classification of Diseases (ICD) codes (Time Period: 2015 – 2018)

Table S4. Percentages of NO_x and $PM_{2.5}$ emissions of each vehicle type within each district of Hong Kong, based on Case Study 1 (i.e., original traffic emissions without adjustments nor incorporation of social characteristics)

Table S5. Traffic NO_x and $PM_{2.5}$ emissions and mortality rates (per 100,000 people) in different case studies, and the percentage of deviation (PD) as calculated by Equation (5) of the main text

Table S6. Comparison of statistical parameters of correlation plots between mortality and emission figures of NO_x and $PM_{2.5}$, by assuming linear regression and least-square fitting holds. Numerical figures in brackets are corresponding statistical parameters for Group 1 districts (G1 – residential districts, N = 7) and Group 2 districts (G2 – non-residential districts, N = 11) respectively, in that order, for Case Studies 3 and 4

Figure S1. Three territories of Hong Kong (i.e., New Territories, Kowloon, and Hong Kong Island) and surrounding areas, with the position of the Hong Kong International Airport included, as extracted from Hong Kong Hopping site

Figure S2. Population figures of 18 districts in Hong Kong

Figure S3. Example of handling long roads within Hong Kong (based on Section 2.3.1(c))

Figure S4. Illustration of Inverse Distance Weighting (IDW) method in calculating traffic emission at position *Y*, which is in between two points, *X* and *Z*, with known emission figures

Table S1. Official demographic and socio quantities of 18 districts in Hong Kong, based on available official sources, with units indicated in brackets and year of attainment afterwards

District (Percentage of Population*)	Population - 2016*	Area (km^2)-2016*	Population Density (per km^2)-2016*	Population Growth (%)-2016**	No. of road segments -2015 #	Geoid Length (m)-2015 #
HONG KONG ISLAND	**1,253,417**	**79.68**	**15,691**	**-1.2**	**4,137**	**451,256**
Central and Western (3.32%)	243,266	12.44	19,391	-2.7	1,498	134,903
Eastern (7.57%)	555,034	18.56	30,861	-2.8	1,025	127,333
Southern (3.75%)	274,994	38.85	7,080	-0.6	687	100,783
Wan Chai (2.46%)	180,123	9.83	17,137	-0.1	927	88,237
KOWLOON	**2,241,347**	**46.93**	**47,748**	**11.0**	**5,950**	**681,878**
Sham Shui Po (5.54%)	405,869	9.35	43,381	11.0	1,212	119,385
Kowloon City (5.71%)	418,732	10.02	41,802	15.5	1,343	175,423
Kwun Tong (8.85%)	648,541	11.27	57,530	10.4	1,047	152,938
Wong Tai Sin (5.80%)	425,235	9.30	45,711	0.4	542	60,074
Yau Tsim Mong (4.68%)	342,970	6.99	49,046	22.3	1,806	154,058
NEW TERRITORIES	**3,840,620**	**952.64**	**4,019**	**7.5**	**9,903**	**1,767,642**
Islands (2.14%)	156,801	175.12	886	14.4	479	285,417
Kwai Tsing (7.10%)	520,572	23.34	22,307	-0.5	1,403	184,081
North (4.30%)	315,270	136.61	2,310	12.3	1,048	215,633
Sai Kung (6.30%)	461,864	129.65	3,563	13.6	823	136,663
Sha Tin (9.00%)	659,794	68.71	9,602	8.6	1,567	204,933
Tai Po (4.15%)	303,926	136.15	2,233	3.5	837	139,879
Tsuen Wan (4.35%)	318,916	61.71	5,149	10.5	853	136,812
Tuen Mun (6.68%)	489,299	82.89	5,894	-2.5	1,013	163,154
Yuen Long (8.29%)	607,200	138.46	4,435	15.0	1,880	301,071

* Based on raw data obtained from “Population by Census 2016” – Percentage of Population Figures are corrected to 2 decimal places; Population Density figures are corrected to nearest km^2.

**All figures are corrected to 1 decimal place, and are obtained by comparing the population figures in 2016 with that of 2006.

The number of road segments are obtained via geo-processing procedures conducted in ArcGIS, while all figures of Geoid Length are corrected to nearest m. The road network data of all 18 districts are obtained from DATA.GOV.HK.

Table S2. Exact number of roads in each district within Case Studies 1 – 4, based on geo-processing procedures in ArcGIS platform, and further spatial considerations, for assessments of NO_x and $PM_{2.5}$ emissions respectively

District (Percentage reduction of roads – Case Study 4 versus Case Study 1)*	Case Study 1 NO_x ($PM_{2.5}$)	Case Study 2 NO_x ($PM_{2.5}$)	Case Study 3 NO_x / $PM_{2.5}$#	Case Study 4 NO_x / $PM_{2.5}$#
HONG KONG ISLAND	**4,014 (4,008)**	**3,821 (3,821)**	**4,008**	**3,246**
Central and Western (18.35%)	1,444 (1,443)	1,346 (1,369)	1,443	1,179
Eastern (23.39%)	992 (991)	945 (965)	991	760
Southern (20.15%)	665 (663)	650 (641)	663	531
Wan Chai (15.01%)	913 (911)	880 (846)	911	776
KOWLOON	**5,759 (5,743)**	**5,354 (5,385)**	**5,743**	**4,213**
Sham Shui Po (33.52%)	1,223 (1,223)	1,098 (1,098)	1,223	813
Kowloon City (21.73%)	1,275 (1,272)	1,216 (1,216)	1,272	998
Kwun Tong (26.65%)	953 (950)	892 (892)	950	699
Wong Tai Sin (28.04%)	535 (527)	480 (480)	527	385
Yau Tsim Mong (25.66%)	1,773 (1,771)	1,668 (1,699)	1,771	1,318
NEW TERRITORIES	**9,509 (9,472)**	**9,025 (9,025)**	**9,472**	**5,041**
Islands (50.54%)	370 (365)	356 (356)	365	183
Kwai Tsing (48.94%)	1,373 (1,365)	1,336 (1,336)	1,365	701
North (34.40%)	965 (965)	914 (914)	965	633
Sai Kung (39.72%)	773 (765)	732 (732)	765	466
Sha Tin (50.39%)	1,538 (1,522)	1,421 (1,421)	1,522	763
Tai Po (48.16%)	814 (814)	769 (769)	814	422
Tsuen Wan (43.74%)	839 (839)	783 (783)	839	472
Tuen Mun (42.12%)	1,002 (1,002)	951 (951)	1,002	580
Yuen Long (55.26%)	1,835 (1,835)	1,763 (1,763)	1,835	821

* All numerical figures are corrected to 2 decimal places.
# All districts have the same number of roads for NO_x and $PM_{2.5}$ assessments for Case Study 3 and 4.

Table S3. Averaged death rates of each district (per 100,000 people) in terms of diseases induced by NO_x and $PM_{2.5}$ traffic emissions. The respective column heading represents the International Classification of Diseases (ICD) codes (Time Period: 2015 – 2018) #

District	A15-A16 **	C33-C34 **	E10-E14 *	I20, I23-I25 *	I26 *	I60-I69 *	J00-J06,J30-J39, J60-J98 **	J12-J18 *	J20-J22 *	J40-J47 **
HONG KONG ISLAND										
Central and Western	*1.92*	*61.23*	6.04	40.64	0.27	66.17	*17.85*	121.91	0.14	*26.91*
Eastern	*2.04*	*77.17*	8.21	46.35	0.54	63.20	*12.71*	180.42	0.06	*27.88*
Southern	*2.13*	*82.55*	10.29	47.42	1.00	69.88	*25.84*	161.96	0.13	*37.64*
Wan Chai	*1.77*	*55.48*	6.30	37.58	0.59	55.09	*13.18*	137.52	---	*19.48*
KOWLOON										
Sham Shui Po	*3.03*	*60.94*	10.69	41.16	1.01	66.41	*8.92*	131.82	0.08	*28.28*
Kowloon City	*2.28*	*63.71*	8.29	42.42	1.06	59.56	*9.59*	209.82	0.16	*26.57*
Kwun Tong	*4.93*	*88.34*	7.39	47.12	0.98	57.64	*15.71*	144.44	0.1	*37.62*
Wong Tai Sin	*3.23*	*95.99*	10.02	58.05	1.1	68.7	*11.91*	203.01	0.08	*41.96*
Yau Tsim Mong	*4.24*	*82.38*	13.61	47.90	1.01	86.22	*14.02*	240.70	0.2	*31.76*
NEW TERRITORIES										
Islands	*1.08*	*46.52*	5.41	33.10	0.65	34.62	*8.44*	84.81	0.22	*16.88*
Kwai Tsing	*3.80*	*81.90*	10.56	47.93	1.11	62.56	*12.85*	192.52	0.13	*30.56*
North	*2.89*	*65.93*	8.03	36.50	0.96	52.87	*10.49*	148.77	0.11	*34.68*
Sai Kung	*2.32*	*54.97*	5.58	23.03	1.01	35.27	*9.05*	94.29	---	*15.06*
Sha Tin	*1.71*	*68.70*	6.92	32.40	0.65	45.23	*16.15*	96.64	0.10	*21.96*
Tai Po	*2.42*	*66.41*	8.80	41.89	1.43	57.39	*12.64*	133.92	---	*32.44*
Tsuen Wan	*2.37*	*58.80*	10.00	37.95	0.86	58.37	*13.12*	193.29	0.11	*23.97*
Tuen Mun	*1.65*	*70.12*	8.92	40.34	1.51	57.49	*11.53*	123.35	0.14	*24.56*
Yuen Long	*2.17*	*61.19*	7.92	33.58	0.81	46.11	*9.44*	108.44	0.16	*24.41*

# The datasets are obtained from HealthyHK, Department of Health, HKSAR Government, and represent the averaged figures throughout the recent 4 years.

* Potential diseases induced by $PM_{2.5}$ traffic emission (i.e., not applicable for NO_x) – in italic representation: E10-E14: Diabetes mellitus; I20, I23-I25: Other ischaemic heart diseases; I26: Pulmonary embolism; I60-I69: Cerebrovascular diseases; J12-J18: Pneumonia; J20-J22: Other acute lower respiratory infections.

** Potential diseases induced by both $PM_{2.5}$ and NO_x traffic emission, including A15-A16: Respiratory tuberculosis; C33-C34: Malignant neoplasm of trachea, bronchus and lung; J00-J06, J30-J39, J60-J98: Remainder of diseases of the respiratory system; and J40-J47: Chronic lower respiratory diseases.

Table S4. Percentages of NO_x and $PM_{2.5}$ emissions of each vehicle type within each district of Hong Kong, based on Case Study 1 (i.e., original traffic emissions without adjustments nor incorporation of social characteristics)

(a) NO_x Emissions (Case Study 1)

District	Private Car (%)*	Taxi (%)*	SPB (%)*	LV (%)*	LGV (%)*	MGV+HGV (%)*	PLB (%)*	Bus (%)*
HONG KONG ISLAND								
Central and Western	2.75	14.45	12.10	2.99	6.73	7.97	5.22	47.79
Eastern	2.79	7.18	9.86	4.69	10.69	14.36	4.29	46.14
Southern	2.44	5.46	11.76	2.86	7.77	7.80	8.59	53.33
Wan Chai	3.92	12.52	12.79	3.80	7.65	10.12	4.75	44.45
KOWLOON								
Sham Shui Po	2.47	6.53	6.97	5.91	16.56	23.49	3.79	34.27
Kowloon City	3.51	10.29	9.33	6.49	13.66	16.02	6.16	34.53
Kwun Tong	3.02	6.62	6.60	5.70	14.09	22.55	7.33	34.09
Wong Tai Sin	2.90	6.25	7.68	4.67	11.08	18.68	7.82	40.92
Yau Tsim Mong	2.76	9.57	10.55	6.64	14.41	16.05	4.95	35.07
NEW TERRITORIES								
Islands	1.44	6.15	12.00	1.47	6.57	19.06	0	53.35
Kwai Tsing	1.41	2.98	5.63	3.03	10.12	45.89	4.74	26.18
North	2.55	3.11	6.84	4.09	10.36	35.40	5.09	32.56
Sai Kung	3.01	4.26	6.44	4.08	10.18	41.01	6.64	24.37
Sha Tin	2.58	3.45	8.64	4.58	11.11	24.99	3.99	40.67
Tai Po	3.82	3.17	6.88	5.99	15.20	36.44	4.17	24.34
Tsuen Wan	2.13	2.59	4.91	3.82	12.36	46.22	2.68	25.30
Tuen Mun	1.39	1.51	3.87	2.68	9.42	59.37	1.99	19.76
Yuen Long	2.06	2.09	7.86	2.72	11.61	44.41	3.59	25.67

SPB: Private light bus and Non-franchised Buses; LV: Van-typed Light Goods Vehicle with less than 3.5 tonnes; LGV: Light Goods Vehicles with 2-axles; MGV+HGV: Medium Goods Vehicles with longer than 6 m; PLB: Green and Red Minibuses

* All numerical figures are corrected to 2 decimal places.

(b) $PM_{2.5}$ Emissions (Case Study 1)

District	Private Car (%)*	Taxi (%)*	SPB (%)*	LV (%)*	LGV (%)*	MGV+HGV (%)*	PLB (%)*	Bus (%)*
HONG KONG ISLAND								
Central and Western	6.94	0	14.73	6.23	7.09	11.54	17.12	36.35
Eastern	5.44	0	10.94	8.45	9.92	18.80	13.08	33.37
Southern	5.54	0	12.55	5.22	7.20	9.92	24.27	35.30
Wan Chai	9.39	0	14.89	7.38	7.23	13.64	14.66	32.81
KOWLOON								
Sham Shui Po	4.08	0	7.40	10.93	14.92	28.14	9.39	25.13
Kowloon City	6.38	0	9.85	11.89	12.03	18.65	16.52	24.68
Kwun Tong	5.37	0	6.55	9.84	11.92	25.86	18.06	22.40
Wong Tai Sin	4.82	0	7.75	8.49	9.26	20.98	21.32	27.38
Yau Tsim Mong	5.24	0	11.03	12.32	13.22	19.71	13.39	25.08
NEW TERRITORIES								
Islands	2.65	0	14.70	2.71	7.36	25.65	0	46.92
Kwai Tsing	1.79	0	4.93	4.66	7.82	51.93	12.23	16.65
North	3.88	0	7.16	6.69	9.06	37.99	12.67	22.55
Sai Kung	4.77	0	5.97	6.54	7.85	46.08	13.95	14.85
Sha Tin	3.85	0	9.63	7.85	9.47	27.31	11.81	30.08
Tai Po	5.28	0	6.88	9.28	12.81	38.54	10.61	16.61
Tsuen Wan	3.12	0	4.75	6.51	10.46	51.81	6.30	17.07
Tuen Mun	2.01	0	3.64	4.21	7.57	65.89	4.55	12.13
Yuen Long	2.91	0	8.29	4.21	10.13	48.86	7.99	17.63

SPB: Private light bus and Non-franchised Buses; LV: Van-typed Light Goods Vehicle with less than 3.5 tonnes; LGV: Light Goods Vehicles with 2-axles; MGV+HGV: Medium Goods Vehicles with longer than 6 m; PLB: Green and Red Minibuses

* All numerical figures are corrected to 2 decimal places.

Table S5. Traffic NO_x and $PM_{2.5}$ emissions and mortality rates (per 100,000 people) in different case studies, and the percentage of deviation (PD) as calculated by Equation (5) of the main text

(a) The case of NO_x

District	Case Study 1 Emission (10^9g/km)*	Case Study 2 Emission (10^9g/km)*	Case Study 3 Emission (10^9g/km)*	Case Study 4 Emission (10^9g/km)*	Mortality Rate (per 100,000 people)*	Diff.**	PD (%)*
HONG KONG ISLAND							
Central and Western	2.79	0.86	1.70	1.55	107.91	– 4.48	– 4.15
Eastern	2.84	1.22	2.55	1.81	119.80	– 1.00	– 0.83
Southern	2.37	0.85	1.99	1.82	148.16	26.99	18.22
Wan Chai	1.73	0.53	1.12	0.92	89.91	– 2.37	– 2.64
KOWLOON							
Sham Shui Po	2.47	0.61	1.38	1.18	101.17	0.56	0.55
Kowloon City	2.44	0.65	1.42	1.29	102.15	– 2.01	– 1.97
Kwun Tong	3.90	1.70	2.74	2.23	146.60	12.07	8.23
Wong Tai Sin	2.13	0.77	2.19	1.79	153.09	32.87	21.47
Yau Tsim Mong	4.22	1.26	2.35	2.25	132.40	– 2.80	– 2.11
NEW TERRITORIES							
Islands	1.14	0.34	0.77	0.25	72.92	2.32	3.18
Kwai Tsing	5.03	2.00	3.21	2.12	129.11	– 1.86	– 1.44
North	3.08	1.07	2.28	1.77	113.99	– 5.72	– 5.02
Sai Kung	1.87	0.54	1.24	0.66	81.40	– 2.47	– 3.03
Sha Tin	3.41	1.48	2.01	1.69	108.52	– 8.37	– 7.72
Tai Po	3.38	1.12	1.82	1.56	113.91	1.20	1.05
Tsuen Wan	2.78	0.86	1.85	1.23	98.26	– 3.87	– 3.94
Tuen Mun	3.30	0.91	1.84	1.63	107.86	– 7.32	– 6.79
Yuen Long	5.00	1.24	2.34	2.12	97.21	– 33.74	– 34.71

* All numerical figures are corrected to 2 decimal places.

** Difference (Diff.) is defined as $MR_P - MR_A$, where MR_P and MR_A are the predicted and actual mortality rates (per 100,000 people) respectively.

(b) The case of $PM_{2.5}$

District	Case Study 1 Emission (10^7g/km)*	Case Study 2 Emission (10^7g/km)*	Case Study 3 Emission (10^7g/km)*	Case Study 4 Emission (10^7g/km)*	Mortality Rate (per 100,000 people)*	Diff.**	PD (%)*
HONG KONG ISLAND							
Central and Western	7.78	3.06	6.39	4.55	343.08	– 10.18	– 2.97
Eastern	11.23	5.07	9.26	5.57	418.58	36.97	8.83
Southern	13.38	3.68	7.91	5.38	438.84	62.52	14.25
Wan Chai	7.22	1.45	3.32	2.75	326.99	23.82	7.29
KOWLOON							
Sham Shui Po	8.43	2.64	6.20	3.65	352.34	24.23	6.88
Kowloon City	13.71	4.13	8.72	6.24	423.46	23.19	5.48
Kwun Tong	18.43	4.84	10.30	6.22	404.27	4.31	1.07
Wong Tai Sin	10.68	4.69	9.56	6.87	494.05	76.21	15.42
Yau Tsim Mong	15.44	5.83	11.50	8.11	522.04	69.54	13.32
NEW TERRITORIES							
Islands	2.84	0.73	1.42	0.62	231.73	– 11.94	– 5.15
Kwai Tsing	31.07	7.30	15.40	9.10	443.92	– 36.21	– 8.16
North	11.18	3.41	7.27	3.96	361.23	24.35	6.74
Sai Kung	6.60	1.45	3.27	1.64	240.58	– 31.50	– 13.09
Sha Tin	10.45	3.90	8.29	4.82	290.46	– 70.41	– 24.24
Tai Po	10.44	5.08	8.13	4.57	357.34	3.55	0.99
Tsuen Wan	20.24	6.77	10.23	6.05	398.84	3.68	0.92
Tuen Mun	15.26	7.10	10.75	6.28	339.61	– 61.78	– 18.19
Yuen Long	16.97	4.83	10.23	7.11	294.23	–130.48	– 44.35

* All numerical figures are corrected to 2 decimal places.
** Difference (Diff.) is defined as $MR_P - MR_A$, where MR_P and MR_A are the predicted and actual mortality rates (per 100,000 people) respectively.

Table S6. Comparison of statistical parameters of correlation plots between mortality and emission figures of NO_x and $PM_{2.5}$, by assuming linear regression and least-square fitting holds. Numerical figures in brackets are corresponding statistical parameters for Group 1 districts (G1 – residential districts, $N = 7$) and Group 2 districts (G2 – non-residential districts, $N = 11$) respectively, in that order, for Case Studies 3 and 4

	Mortality Rate (y) against Traffic Emission (x) **NO_x**	Mortality Rate (y) against Traffic Emission (x) **$PM_{2.5}$**
**Case Study 1**	Best-fit line: $y = 7.88 \times 10^{-9}x + 88.86$<br>$R$ value: 0.371<br>RMSE: 21.5<br>p value: 0.129<br>F-stat: 2.56	Best-fit line: $y = 6.38 \times 10^{-7}x + 289.16$<br>$R$ value: 0.509<br>RMSE: 70.7<br>p value: 0.031<br>F-stat: 5.59
**Case Study 2**	Best-fit line: $y = 2.81 \times 10^{-8}x + 84.39$<br>$R$ value: 0.539<br>RMSE: 19.5<br>p value: 0.0208<br>F-stat: 6.58	Best-fit line: $y = 2.48 \times 10^{-6}x + 266.48$<br>$R$ value: 0.593<br>RMSE: 66.1<br>p value: 0.0094<br>F-stat: 8.71
**Case Study 3**	Best-fit line: $y = 2.65 \times 10^{-8}x + 61.27$<br>$R$ value: 0.720 (G1: 0.819, G2: 0.815)<br>RMSE: 16.1 (G1: 13.4, G2: 14.8)<br>p value: 0.000756 (G1: 0.0245, G2: 0.00222)<br>F-stat: 17.2 (G1: 10.1, G2: 17.9)	Best-fit line: $y = 1.62 \times 10^{-6}x + 237.97$<br>$R$ value: 0.674 (G1: 0.777, G2: 0.889)<br>RMSE: 60.7 (G1: 52.8, G2: 39.3)<br>p value: 0.00218 (G1: 0.0399, G2: 0.000253)<br>F-stat: 13.3 (G1: 7.6, G2: 33.9)
**Case Study 4**	Best-fit line: $y = 3.23 \times 10^{-8}x + 62.43$<br>$R$ value: 0.783 (G1: 0.760, G2: 0.850)<br>RMSE: 14.4 (G1: 15.2, G2: 13.5)<br>p value: 0.000121 (G1: 0.0473, G2: 0.000932)<br>F-stat: 25.4 (G1: 6.9, G2: 23.3)	Best-fit line: $y = 2.79 \times 10^{-6}x + 226.32$<br>$R$ value: 0.754 (G1: 0.717, G2: 0.958)<br>RMSE: 54.0 (G1: 58.4, G2: 24.6)<br>p value: 0.000304<br>(G1: 0.0697, G2: 3.56×10^{-6})<br>F-stat: 21.0 (G1: 5.3, G2: 100.0)

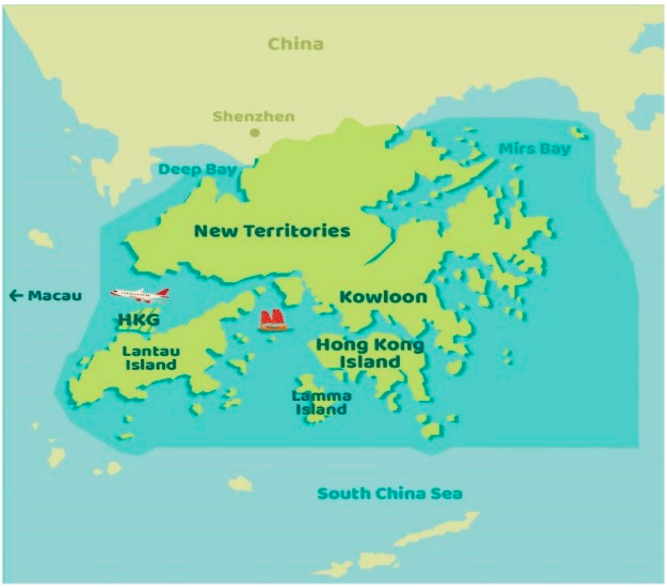

Figure S1. Three territories of Hong Kong (i.e., New Territories, Kowloon, and Hong Kong Island) and surrounding areas, with the position of the Hong Kong International Airport included, as extracted from Hong Kong Hopping site

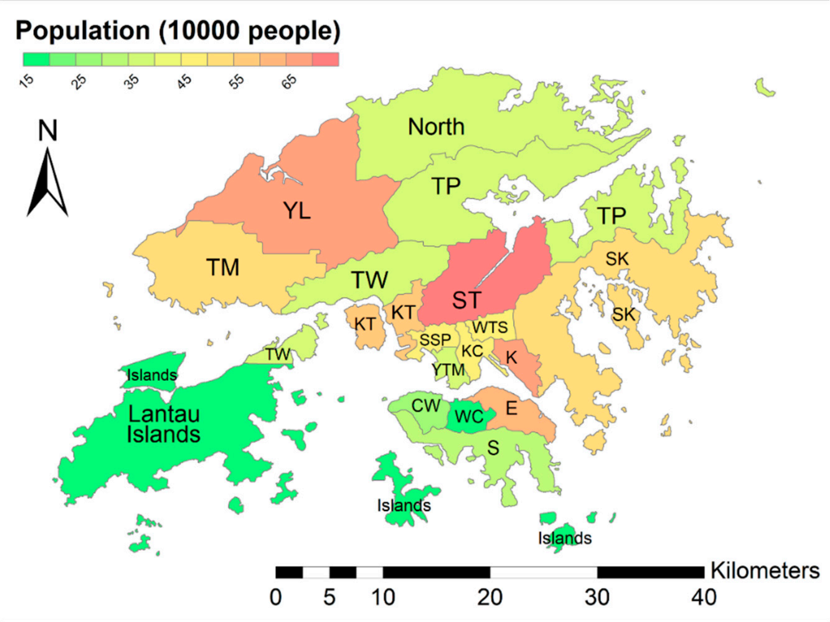

Figure S2. Population figures of 18 districts in Hong Kong. The color bar ranges from 150,000 to 700,000 people. Labels and distribution of districts in 3 territories of Hong Kong are as provided. *New Territories* (from right to left) – Islands; TM: Tuen Mun; YL: Yuen Long; TW: Tsuen Wan; North; TP: Tai Po; ST: Sha Tin; SK: Sai Kung; *Kowloon* (from right to left) – KT: Kwai Tsing; SSP: Sham Shui Po; YTM: Yau Tsim Mong; KC: Kowloon City; WTS: Wong Tai Sin; K: Kwun Tong; *Hong Kong Island* (from right to left) – CW: Central and Western; S: Southern; WC: Wan Chai; E: Eastern

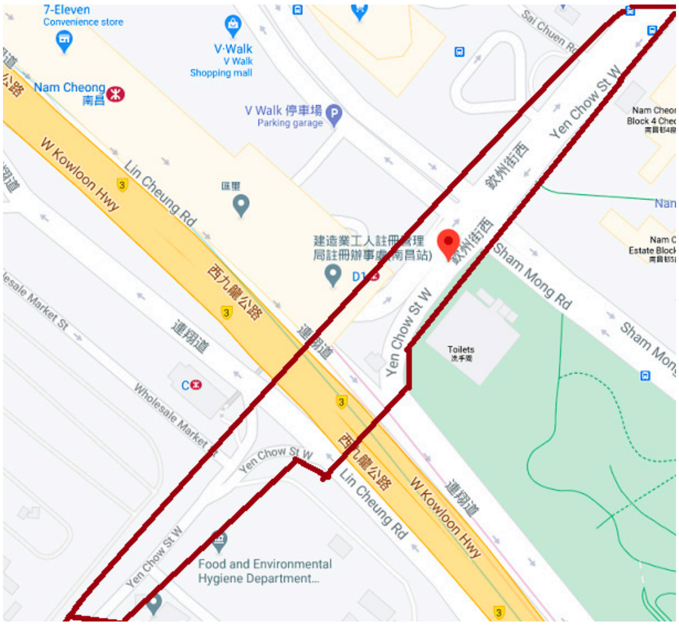

Figure S3. Example of handling long roads within Hong Kong (based on Section 2.3.1(c)).
The spatial location of Yen Chow Street West (surrounded by the brown box, as extracted from Google Map), which passes through two districts of Hong Kong, namely Sham Shui Po and Yau Tsim Mong. Surrounding red symbols represent MTR stations, while blue pointers indicate convenience store, shopping malls and car parks

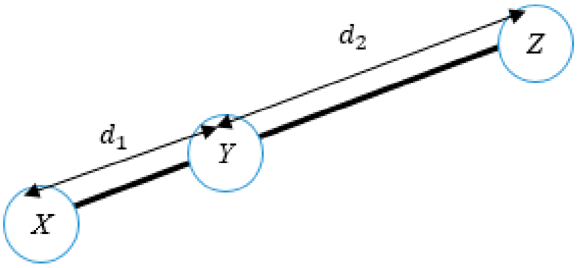

Figure S4. Illustration of Inverse Distance Weighting (IDW) method in calculating traffic emission at position Y, which is in between two points, X and Z, with known emission figures

References

DATA.GOV.HK. Road Network, Transport Department. Available online: https://data.gov.hk/en-data/dataset/hk-td-tis_6-road-network (accessed on 2 Mar 2021).

HealthyHK, Department of Health, HKSAR Government. Death Statistics - by sex, age and residential district, causes of death in tabulation list of the International Classification of Diseases (from 2001 onwards). Available online: https://www.healthyhk.gov.hk/phisweb/enquiry/mo_ysad10_e.html (accessed on 2 Mar 2021).

Hong Kong Hopping. So Where is Hong Kong Exactly. https://www.hongkonghopping.com/location.html (accessed on 27 Apr 2021).

Population by Census, 2016. Main Tables. Available online: https://www.bycensus2016.gov.hk/en/bc-mt.html (accessed on 2 Mar 2021).
